# Properties and Applications of Nanoparticles from Plant Proteins

**DOI:** 10.3390/ma14133607

**Published:** 2021-06-28

**Authors:** Narendra Reddy, Marco Rapisarda

**Affiliations:** 1 Center for Incubation Innovation Research and Consultancy, Jyothy Institute of Technology, Thataguni Post, Bengaluru 560109, India; 2 Institute of Polymers, Composites and Biomaterials, National Research Council, via Paolo Gaifami 18, 95126 Catania, Italy; marcorapis7@gmail.com

**Keywords:** plant proteins, nanoparticles, zein, gliadin, soy proteins, anti-solvation, controlled release, emulsification

## Abstract

Nanoparticles from plant proteins are preferred over carbohydrates and synthetic polymeric-based materials for food, medical and other applications. In addition to their large availability and relatively low cost, plant proteins offer higher possibilities for surface modifications and functionalizing various biomolecules for specific applications. Plant proteins also avoid the immunogenic responses associated with the use of animal proteins. However, the sources of plant proteins are very diverse, and proteins from each source have distinct structures, properties and processing requirements. While proteins from corn (zein) and wheat (gliadin) are soluble in aqueous ethanol, most other plant proteins are insoluble in aqueous conditions. Apart from zein and gliadin nanoparticles (which are relatively easy to prepare), soy proteins, wheat glutenin and proteins from several legumes have been made into nanoparticles. The extraction of soluble proteins, hydrolyzing with alkali and acids, conjugation with other biopolymers, and newer techniques such as microfluidization and electrospraying have been adopted to develop plant protein nanoparticles. Solid, hollow, and core-shell nanoparticles with varying sizes and physical and chemical properties have been developed. Most plant protein nanoparticles have been used as carriers for drugs and as biomolecules for controlled release applications and for stabilizing food emulsions. This review provides an overview of the approaches used to prepare nanoparticles from plant proteins, and their properties and potential applications. The review’s specific focus is on the preparation methods and applications, rather than the properties of the proteins, which have been reported in detail in other publications.

## 1. Introduction

Nanoparticles are one of the most unique entities that enhance performance, extend applications and provide an opportunity to develop materials that can address many major scientific challenges. Nanoparticles made from metals, carbon, organic and inorganic polymers and their blends have been extensively studied and used for medical, environmental, food, energy and other applications [1,2,3,4]. Nanoparticles in various shapes and sizes, including solid, hollow (nanocapsules) and core-shell structures have been developed. Nanoparticles are also classified based on their source, such as polymeric nanoparticles, magnetic nanoparticles, liposomes, carbon nanotubes, quantum dots, dendrimers, metallic nanoparticles, etc. [5,6]. When used for medical applications, nanoparticles have been able to escape detection by the immune system and accumulate in tumors and organs, and are hence considered ideal for targeted drug delivery. The larger surface area and the ability to load entities inside hollow particles provide them with extraordinary loading capacity. Such nanoparticles have also been used for the removal of dyes and chemicals from polluted water.

**Figure 1:**
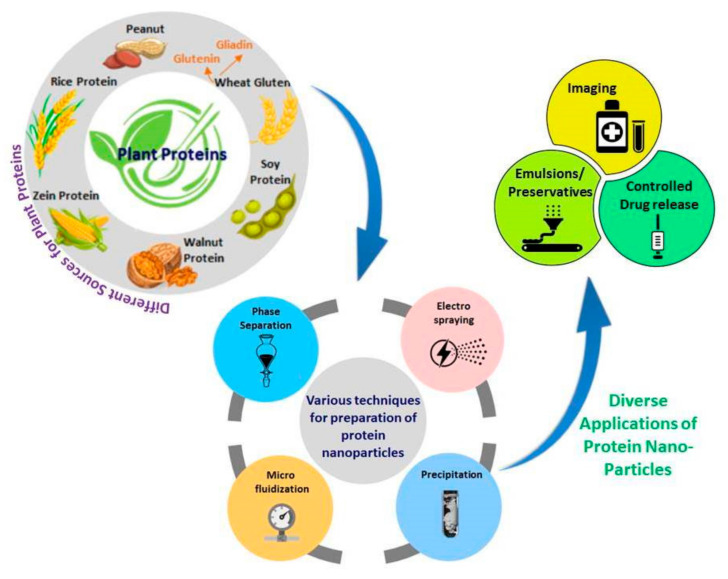
An overview of the sources used, preparation methods, and potential applications of nanoparticles from plant proteins.

Compared to carbohydrates or metals, protein-based nanoparticles have been considered ideal for medical, food, cosmetic, and other applications. Proteins offer the possibility for surface modifications and the attachment of drugs and other biomolecules through covalent, ionic, hydrogen bonding and other associations. Both animal- and plant-based proteins have been extensively studied and made into nanoparticles using different techniques [7,8]. Zein from corn, gliadin and glutenin from wheat gluten, and soy proteins are some of the common plant proteins used for developing nanoparticles. Proteins in lesser-known legumes such as peanuts and chickpeas, and proteins in plant extracts are also considered as sources for nanoparticles. Similarly, animal-based proteins such as collagen, albumin and keratin from feathers and wool have been studied for developing nanoparticles. Fibroin and sericin in silk are also found to provide nanoparticles with excellent biocompatibility, stability, and properties required for medical applications. A few proteins such as zein have inherent fluorescence, which is useful for imaging and detection purposes. Compared to animal proteins, plant proteins do not cause immunogenicity and are hence preferable for developing nanomaterials [9,10]. An overview of the major plant proteins used for developing nanoparticles, the common methods of preparation, and their applications are given in Figure 1.

Although protein-based nanoparticles offer unique properties, there are considerable constraints involved in their preparation and applications. Primarily, most proteins do not dissolve in aqueous solvents or common chemicals, and it is hence difficult to make them into nanoparticles. Using toxic solvents or hydrolyzing the proteins results in the loss of many characteristics. In addition, being biomolecules, the stability and shelf-life of the particles is also a concern, and obtaining uniform particle size or narrow size distribution is also challenging. Nevertheless, the benefits and scope of using protein nanoparticles, particularly those made from plant proteins, outweigh their limitations. Apart from the conventional methods of preparing nanoparticles through co-precipitation or phase separation, newer methods such as atomization and high temperature shearing provide better options to obtain nanoparticles with desired features. Here, we review the methods of preparation, properties and applications of protein-based nanoparticles. Hence, this review excludes animal proteins, other polymeric and non-polymeric sources, liposomes, carbon dots and other miscellaneous sources used to make nanoparticles or nanostructures. We hope that this review will increase research and development of protein-based nanoparticles, particularly from newer sources. We also hope that proteins in edible and non-edible oil seeds, which could be unique sources for nanoparticles, will be explored. Detailed investigations on the toxicity and in vivo applications of protein nanoparticles should also be done to ensure that the uniqueness of plant proteins can be implemented in practice to solve major health and environmental challenges.

## 2. Nanoparticles from Zein

### 2.1. Preparation and Stabilization of Zein Nanoparticles

**Figure 2:**
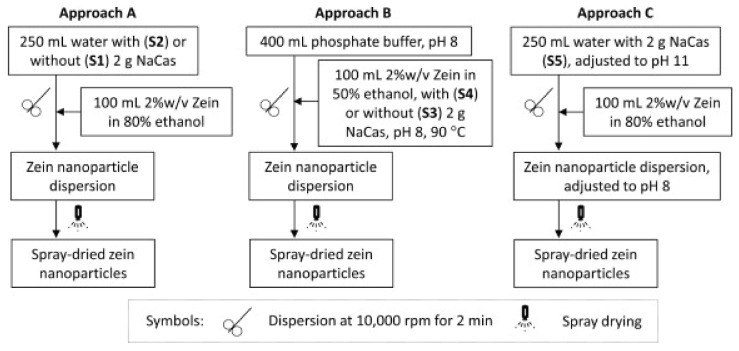
Three different approaches used to prepare zein nanoparticles through spray drying. Reprinted with permission from ref. [15]. Copyright 2014 Elsevier.

Zein, which is soluble in aqueous alcohols, is one of the more prominent plant proteins used for the preparation of nanoparticles for food, medical and other applications [11,12]. Nanoparticles from zein and other proteins have been prepared through several approaches, including protein aggregation in solutions (antisolvent precipitation), emulsion-based phase separation, associative phase separation, electro-spraying, electrodynamic atomization, etc. [13,14]. The conventional approach to preparing zein nanoparticles is to dissolve zein in ethanol and subsequent, precipitation of the proteins by adjusting the pH. However, nanoparticles obtained by this approach are relatively unstable and do not easily disperse in water. To overcome these challenges, a new method of preparing zein nanoparticles by dispersing zein in sodium caseinate and adjusting the pH or ethanol concentrations was studied (Figure 2) [15]. Zein nanoparticles with a diameter of 125 nm were obtained via spray drying, with excellent dispersibility in water, particularly for samples obtained at pH 7.0 and 0–300 mM of NaCl, found. Nanoparticles did not precipitate even after storage for 15 days.

**Figure 3:**
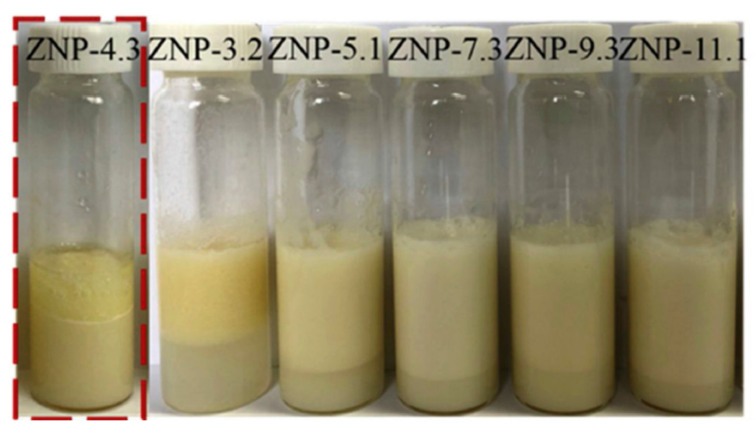
Pickering emulsions containing zein nanoparticles prepared after treating with alkaline pH showed substantially increased stability even after 28 days of storage. Reprinted with permission from ref. [16]. Copyright 2020 Elsevier.

In a relatively simple process, it was reported that adjusting the pH of a zein solution could change the surface properties and help to obtain nanoparticles with a lower size and higher stability. In this study, zein was dissolved in 80% ethanol and the pH was adjusted from 3.2 to 11.1 and incubated for 24 h. The treated zein was later dialyzed and the solution obtained was used to prepare the nanoparticles by co-precipitation in distilled water. The shape, size and properties of the nanoparticles changed substantially depending on the pH. For example, particle size, polydispersity index (PDI), and zeta potential were 58.5 nm, 0.142 and −45 mV at pH 11.1, compared to 430 nm, 0.427 and 27.6 mV at pH 3.2, respectively. Although treating the nanoparticles under alkaline conditions caused increased deamidation, no significant change in molecular weight was observed at the pHs studied. Pickering emulsions, which are difficult to maintain, showed considerable stability even after 28 days, after the addition of alkaline zein nanoparticles (Figure 3) [16]. Combining zein nanoparticles and cinnamon essential oil was also able to stabilize Pickering emulsions, and, further, to replace butter in pound cakes and prevent fungal growth [17]. It was suggested that the aldehyde groups of cinnamaldehyde could react with amino groups in zein and enhance the visco-elastic properties. A combination of 20 g zein nanoparticle solution, 15 g oil and 5 g cinnamon essential oil could replace 20% of butter, improve shelf life, and also lower calorie intake without affecting the color or texture of pound cakes.

**Figure 4:**
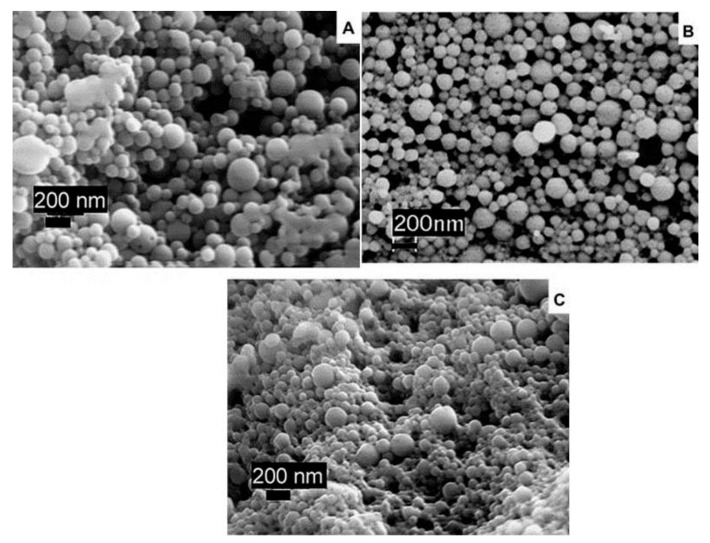
SEM images of zein nanoparticles obtained at three different shear speeds: 5000 rpm (**A**), 10,000 rpm (**B**) and 15,000 rpm (**C**) speeds. Reprinted with permission from ref. [18]. Copyright 2009 Elsevier.

A liquid-liquid dispersion process was proposed to obtain zein nanoparticles for a food grade nanoscale delivery system [18]. In this approach, zein was dissolved in 55–90% aqueous ethanol and the solution was then sheared in deionized water using a homogenizer operating at speeds between 5000 and 15,000 rpm. The size and shape of the nanoparticles varied considerably depending on the shearing speed (Figure 4). The mean diameter of the nanoparticles generally decreased from about 400 to 180 nm as the shear rate increased from 1000 to 3000. The addition of 1% zein nanoparticles into carboxymethyl cellulose substantially modified the viscosity and made them suitable for food applications [18]. A liquid-liquid dispersion and anti-solvent precipitation system was found suitable to combine phytotherapy and nanotechnology to treat fish pathogens [19]. Phytochemicals such as eugenol, garlic essential oil, and a combination of the two were encapsulated in zein nanoparticles having an average diameter of 150 nm, a zeta potential of 30 mV and a PDI of 0.2. An encapsulation efficiency of up to 90% was possible, and the compounds did not show any decrease in activity even after 90 days. The encapsulated nanoparticles displayed antibacterial activity against pathogenic bacteria such as *Aeromonas hydrophila*, *Edwardsiella tarda* and *Steptococcus iniae*, suggesting their suitability in treating fish diseases [19].

Obtaining zein nanoparticles using ethanol as a solvent poses flammability risks and also encapsulates various lipophilic compounds that co-dissolve in ethanol. To avoid this, it was proposed to dissolve zein in nonflammable propylene glycol and to later stabilize the nanoparticles using gum arabic. Particles having a diameter of 250 nm, a height of 51.3 nm, and high stability between pH 3 and 8 were obtained. Gum Arabic was sorbed on the zein due to electrostatic and hydrophobic interactions. Encapsulating (89%) pepper mint oil was possible without affecting particle size or dispersibility. The pepper mint oil could be gradually released from the particles, with 100% release within 72 h at pH 2.0 suggesting their suitability for food applications [20].

The stability of zein nanoparticles in physiological conditions and at various pHs and temperatures is a major concern. Several approaches have been used to improve the stability of zein nanoparticles. Among different choices, the encapsulation of zein with other biopolymers and synthetic materials is a preferred alternative to protect zein and improve the stability and bioavailability of the payloads [21]. A review by Tapia-Hernández lists the various polysaccharides that have been used to encapsulate and provide additional stability to zein nanoparticles. In addition to increasing stability, encapsulation also protects the nanoparticles under various physiological environments and increases bioavailability [22]. A list of zein-polysaccharide nanoparticles with antioxidant properties and which should be considered for treating various chronic degenerative diseases is given in Table 1, and the possible approaches used to stabilize zein nanoparticles using various biomolecules are listed in Table 2.

### 2.2. Zein Nanoparticles for Controlled Release Applications

**Figure 5:**
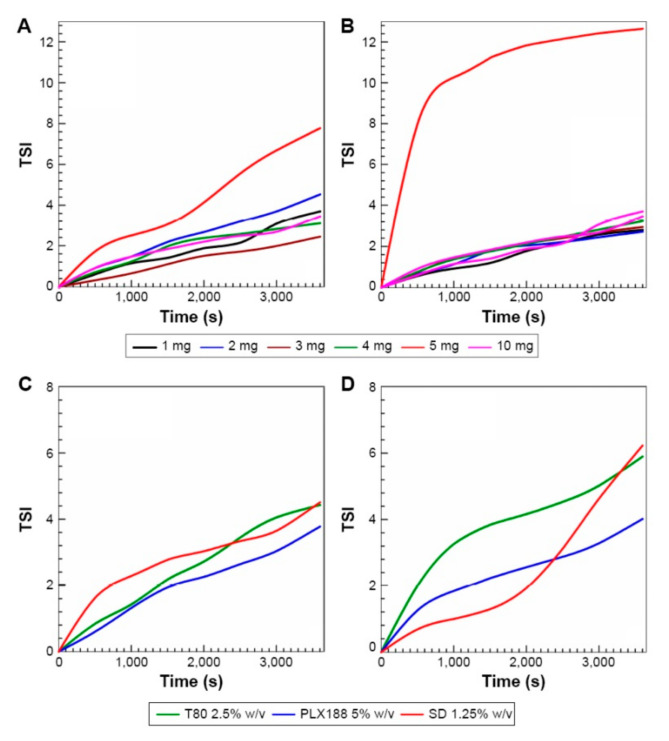
Effect of varying zein concentrations (**A**,**B**) and surfactants (**C**,**D**) on the formation conditions and Turbiscan Stability Index of zein nanoparticles. T 80 is Tween 80, PLX is Poloxamer 188, and SD is sodium deoxycholate monohydrate. Reprinted with permission from ref. [55]. Copyright 2018 Dove Press.

Many natural compounds have been encapsulated using zein in order to enhance the applications and increase the efficiency of the controlled delivery of food, medical and pharmaceutical products. Procyanids found in cranberries and other fruits have antioxidant potential, and are suggested to treat various chronic diseases, including cancer, cardiovascular conditions and urinary tract infections. However, procyanids have low oral bioavailability and hence need to be encapsulated for efficient delivery. Since procyanids are reported to have a high binding affinity to proteins, the feasibility of encapsulating the biomolecule in zein nanoparticles was studied [52]. Depending on the ratio of the zein to the procyanids, the particle size ranged from 382 to 447 nm, and the zeta potential varied from 14 to 22 mV. The loading efficiency also increased up to 75% with mass ratios due to better hydrogen bonding and hydrophobic interactions. When incubated with human promyelocytic leukemia Human Leukemia (HL-60 cells), the encapsulated nanoparticles showed considerably lower cytotoxicity compared to pure procyanids [52]. Up to 100% encapsulation efficiency of *Hibiscus sabdariffa* onto zein nanoparticles with sizes between 216 and 289 nm and a PDI between 0.25 and 0.38 was reported. The encapsulated particles were stable for up to six months without major changes in diameter or to the PDI [53]. In another report, a comparative study was done to determine the advantages of using zein for encapsulating Rutin, a polyphenolic bioflavanoid, rather than poly(lactic acid-co-glycolic acid) (PLGA). Particles were formed using the anti-solvent approach, in the presence of non-ionic or anionic detergents. The zein nanoparticles obtained had an average diameter of 130 nm, which did not change due to the addition of rutin. A loading capacity of up to 18% was possible on zein compared to 0.5% for the PLGA particles. The drug had a desirable release profile without any cytotoxicity. The antioxidant activity of rutin was preserved even at the intra-cellular localization level [54]. In a similar study, the possibility of obtaining stable zein nanoparticles by combining them with different surfactants for the potential delivery of various compounds was investigated [55]. The effect of conditions such as the pH, temperature, entrapment efficiency, and the Turbiscan Stability Index (TSI) during nanoparticle preparation, on the size and properties of the nanoparticles were studied. Nanoparticles with diameters between 100 and 200 nm were obtained depending on the conditions used during preparation (Figure 5). Among the different conditions studied, nanoparticles obtained using sodium deoxycholate monohydrate (1.25%) provided better stability, an average size of 100 nm and a zeta potential of −30 mV. The particles were stable even after heating at 50 °C and after incubating with phosphate buffered saline (PBS). However, surfactants increased the cytotoxicity of the nanoparticles above a certain concentration [54].

The coating of zein nanoparticles with chitosan increased the particle size to 500 nm from 300 nm, and the zeta potential changed to +24 mV from −30 mV. When used for loading retinol, chitosan coating also increased the encapsulation efficiency from 65% to 80% and provided considerably improved release rates and photochemical stability [56]. Similar to chitosan, zein nanoparticles were coated with alginate and gelatin through electrostatic deposition. Such coating not only improved the stability of the nanoparticles, but also prevented aggregation between a pH of 3 and 7 without affecting the thermal stability. The coated nanoparticles did not show any appreciable change even after being heated at 80 °C for 40 min in 10% sucrose solution. Compared to 8% before coating, the bioavailability increased to 22% after coating when the nanoparticles were exposed to a simulated gastrointestinal tract. A considerable increase in antioxidant activity and ferric ion reducing power was also observed due to the coating on the zein nanoparticles [57]. Although coating may improve the efficiency of the zein nanoparticles for drug delivery, it should be ensured that such coatings are biocompatible, inexpensive and easily soluble. Coating using techniques such as electrostatic deposition may not be feasible for large scale production.

The extent of loading, and the release of bioactive compounds and other entities from zein nanoparticles also depends on the mode of association, the physical and chemical interactions between the particles, and the payloads they carry. For instance, chemical interactions occurred when folic acid was combined chemically with zein through the covalent links between the carboxyl group of folic acid and the amino groups in zein. Alternatively, physical interaction occurred when zein and folic acid were dissolved in 70% ethanol in the presence of lecithin and pluoronic F127 solution and passed through a microfluidizer. Later, the ethanol was evaporated, the solution was dialyzed to remove the surfactant, and the proteins were freeze-dried to obtain the dry nanoparticles. Folic acid-containing nanoparticles prepared through covalent crosslinking had a higher loading capability and a slower and more sustained release compared to the physically loaded folic acid. However, nanoparticles prepared through both of the methods were biocompatible and did not cause any cytotoxicity to HeLa cells. Overall, covalent crosslinking was suggested to be a desirable and more appropriate method for obtaining sustained release and targeted delivery [58].

### 2.3. Curcumin Delivery through Zein Nanoparticles

Curcumin is a natural compound that has been reported to have antimicrobial, anticancer, and many other medicinal properties. However, curcumin is insoluble in water and hence presents challenges for its use in medical applications, particularly for controlled release applications. Hence, various carriers have been used to deliver curcumin in vitro and in vivo and for food applications. For example, the possibility of using zein nanoparticles to deliver curcuminoids as nutraceuticals for oral administration was studied. Nanoparticles were prepared with 6, 10, or 15% curcuminoids, and the effect of administering the curcumin-loaded zein into Wastar rats (250 mg/kg of body mass) was studied. Blood and plasma were collected from the mice at different intervals and the bioavailability was checked. Up to 98% encapsulation efficiency and 9.2% loading capacity was possible, and the curcumin loaded onto the zein nanoparticles showed considerable stability and were well-tolerated [59]. More importantly, a 9 times higher bioavailability value was observed for the zein nanoparticles which contained curcumin compared to pure curcumin. The curcumin could also be encapsulated onto zein after being coating with carboxylic curdlans with three different chain configurations. The pure particles developed had an average diameter of 183 nm, a zeta potential of 17.7 mV, and an encapsulation efficiency of 67%. The modified nanoparticles showed substantial differences in properties, as seen from Table 3, due to the hydrogen bonding, the hydrophobic and electrostatic interactions between curcumin, and the zein and carboxylic curdlans. The curcumin in the nanoparticles showed considerable antioxidant activity and easy redispersibility, and hence is considered suitable for food, cosmetic, and other applications [60]. The zein nanoparticles stabilized with sulfated anionic polysaccharide-dextran sulfate were stable at different pHs and heats, and showed stability even after longer storage times. Dextran sulfate coating also reduced the hydrophobicity and did not show any toxicity to the colonic epithelial cells. The treated nanoparticles were able to encapsulate up to 85% of the curcumin and improved bioavailability when used for controlled release applications [61].

Zein nanoparticles intended as carriers for curcumin were stabilized with k-carrageenan, a polysaccharide, and Tween 80, a surfactant. The addition of the polysaccharide reduced the particle size, increased the PDI (<0.20), but enhanced the thermal stability. Up to 34% encapsulation efficiency was possible, which is relatively low compared to data reported in other publications [62]. In a unique approach, zein nanoparticles were coated with a film of tannic acid in the presence of CuCl_2_ or FeCl_3_ solutions [39]. The extent of film formed was dependent on the pH and the multivalent level of coordination bonding. After loading with doxorubicin, nanoparticles with an average diameter of 179 nm for Cu^II^ and 186 nm for FE^III^ modified nanoparticles were obtained without any cytotoxicity against HepG2 cells. The zein nanoparticles were able to convert Au^3+^ ions into Au and substantially enhanced the surface plasmon resonance. These complex nanoparticles were suggested to be suitable for photothermal therapy for treating cancer [39].

### 2.4. Controlled Release of Drugs Using Zein Nanoparticles

**Figure 6:**
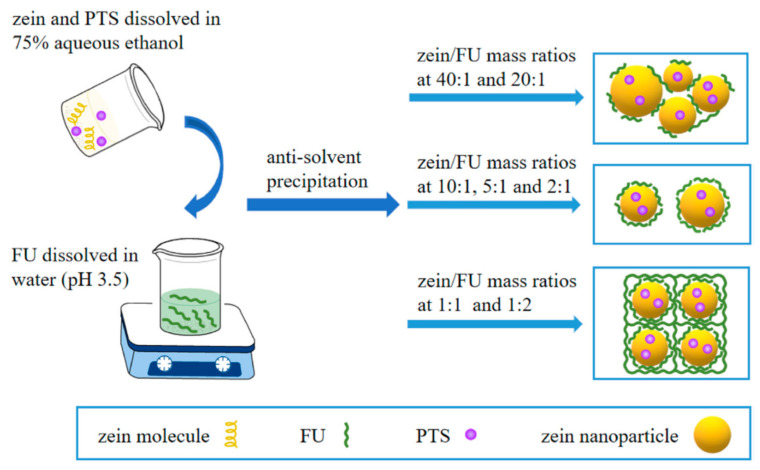
Schematic representation of the preparation of and possible interactions between zein, fucoidan (FU) and pterostilbene (PTS). Reprinted with permission from ref. [64]. Copyright 2020 Elsevier.

**Figure 7:**
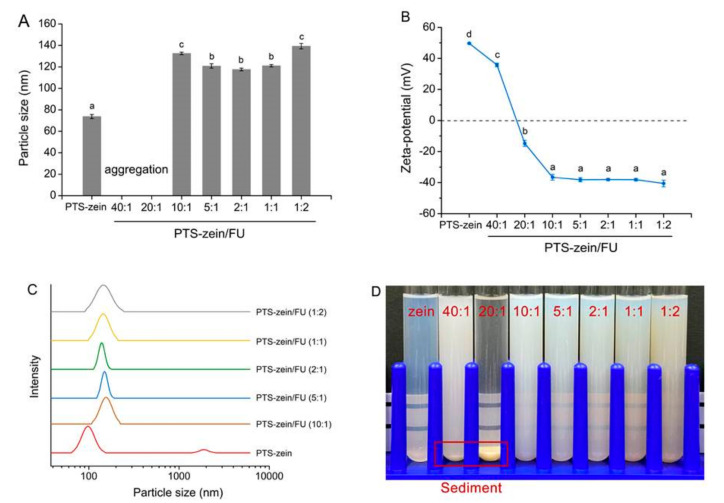
Properties of the composite zein-Fu nanoparticles with and without pterostilbene (PTS). Changes in the nanoparticle size (**A**), zeta potential (**B**), size variation with and without PTS (**C**), and the appearance of the nanoparticles (**D**). Reprinted with permission from ref. [64]. Copyright 2020 Elsevier.

Maytansine (DM1) is a potent anticancer drug, but it is difficult to administer due to its poor solubility in water and its toxic side effects. Hence, targeted delivery of the drug using zein nanoparticles was evaluated. Nanoparticles with a diameter of 112 nm, a zeta potential of 37 mV, and a PDI of 0.2 were considered to be suitable for in vivo drug delivery. An encapsulation efficiency of 83% and drug loading of 3.3% was achieved. The drug containing nanoparticles showed a strong inhibition of A549 cells and up to 97% tumor inhibition rates without causing toxicity, and were found to accumulate in the tumor [63]. Composite nanoparticles of zein and fucoidan (FU) were developed as a potential carrier for pterostilbene (PTS), a nutraceutical with multiple benefits. The biomolecule was encapsulated into the nanoparticle composite, obtained through the anti-solvent precipitation approach, with varying ratios of zein to fucoidan: between 1:1 and 10:1. The size of the nanoparticles was between 120 and 150 nm, and FTIR analysis showed that the nanoparticles were mainly formed through electrostatic, hydrogen bonding and hydrophobic interactions between the three entities involved (Figure 6). Some of the properties of the composite nanoparticles are shown in Figure 7. Encapsulation efficiencies ranged between 27 and 90%, and the loading capacity was between 2.5 and 7.9% depending on the ratio of PTS-zein to FU. Encapsulation increased the photochemical stability of PTS, and a uniform controlled release was possible under simulated gastrointestinal conditions. The nanoparticles did not show any toxicity towards Caco-2, HK-2 or L-O2 cells, suggesting their suitability for controlled delivery [64]. Another biomolecule resveratrol (RSV) was also loaded onto zein nanoparticles with sizes between 120 and 180 nm, a PDI of <0.150, and a zeta potential of +20 mV. Sucrose used as a cryoprotectant increased shelf-life and also improved the redispersion of the nanoparticles in water. The nanoparticles were able to protect RSV against degradation by the intestinal cells and allowed for the permeability of RSV across CaCO_2_ and CaCO_2_/HT29-MTX cell monolayers [65].

As another possibility, zein was hybridized with chondroitin sulfate (CS) in different ratios and made into nanoparticles with an average diameter of 158 nm, through the solvent displacement method. The nanoparticles were loaded with docetaxel with an encapsulation efficiency of 64.2%. The drug showed sustained release for up to 72 h following first-order kinetics. The modified nanoparticles were stable in serum and had considerably lower IC_50_ values compared to free docetaxel, and enhanced accumulation in tumors and a nearly 10 times longer terminal half-life compared to the free drug—suggesting their suitability for treating cancer—was also observed [66]. CS-loaded zein nanoparticles could be further stabilized depending on the pH and the stage of addition of CS. Nanoparticles with an average size of 197 nm and a zeta potential of −48 mV were obtained. The particles obtained at pH 7 showed considerable stability compared to those obtained at pH 3. CS was able to form macromolecular complexes with zein through non-electrostatic interactions when added before the anti-solvent process at pH 7, and this is hence preferable to adding zein after the particle formation [67]. With diameters between 158 and 397 nm, the nanoparticles were able to encapsulate up to 6.1 mg of DNA per gram of zein, with an encapsulation efficiency of 65%. Encapsulation by zein protected the DNA from degradation and also provided sustained release for up to 7 days, suggesting the nanoparticles’ suitability for oral gene delivery, intramuscular delivery, and for developing tissue-engineering scaffolds [68]. Although extensive studies have been conducted regarding the use of zein as a carrier for drugs and other pharmaceuticals, the amount of payload that can be loaded onto zein is limited and may not be sufficient for treating tumors or other diseases. Similarly, the ability of the zein nanoparticles to escape detection by the immune system and to deliver payloads to targeted organs needs to be investigated further.

### 2.5. Zein Nanoparticles for Imaging

**Figure 8:**
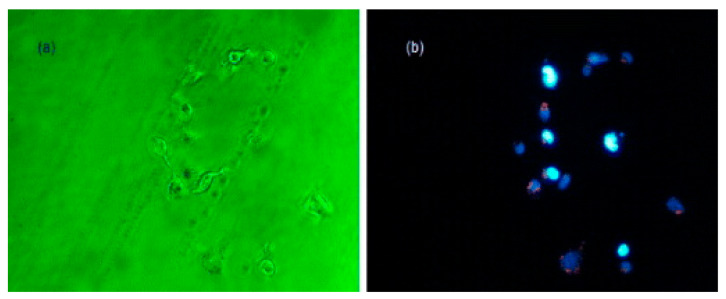
Quantum dot-containing zein nanoparticles were able to internalize and emit fluorescence useful for imaging as seen from a fluorescent (**a**) and confocal image (**b**). Reprinted with permission from ref. [69]. Copyright 2012 IOP Science.

**Figure 9:**
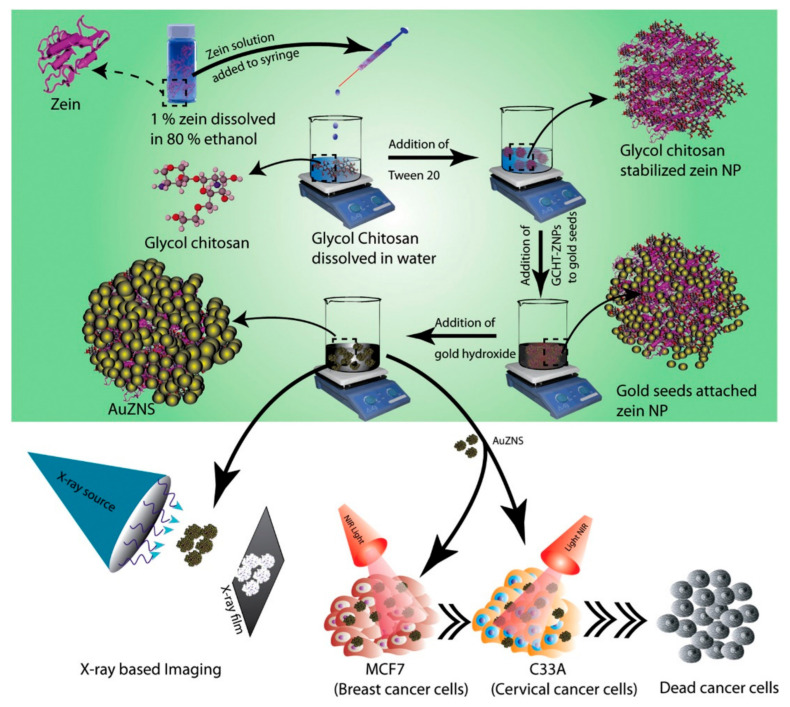
Process of preparing the zein-chitosan gold coated nanoparticles (nanoshells) (NP) for imaging and phototherapy applications. Reprinted with permission from ref. [70]. Copyright 2018 Elsevier.

Zein is reported to have inherent fluorescent properties, which are desirable for imaging applications. Furthermore, additional fluorescence can be imparted by adding dyes and quantum dots. In one such attempt, zein nanoparticles were prepared through the liquid-liquid dispersion technique, followed by freeze-drying. Quantum dots were also prepared using zinc acetate and manganese sulfate, which were added into the zein solution. Later, 5-fluororocil, an anticancer drug, was conjugated onto the fluorescent nanoparticles, which showed an absorbance between 220 and 350 nm. The diameter of pure nanoparticles was between 600 and 700 nm, which increased to between 800 and 900 nm after being loaded with the drugs. The quantum dots were 5 nm in diameter and could be easily loaded onto the zein nanoparticles. The drugs loaded onto the nanoparticles had an encapsulation efficiency of 60% and a sustained release (70%) was possible in about 8 h. Zein- and quantum dot-containing particles had high cell viability for MCF7 breast cancer cells and L929 cells, but drug-loaded nanoparticles showed considerable cytotoxicity for both the cell lines [69]. Further, the internalized nanoparticles retained and emitted the fluorescence at detectable levels (Figure 8), and are hence considered suitable for imaging purposes. Zein-glycol chitosan complex nanoparticles (nanoshells) were developed using the anti-solvent precipitation method. These nanocomplexes were conjugated with folic acid or attached with gold seeds to make them suitable for imaging and for enhanced targeted delivery for treating cancer [70]. A schematic of the process used for preparing these nanoparticles is shown in Figure 9. The nanocomplexes were biocompatible and non-toxic even at considerably high dosages, responded to photothermal therapy, and acted as contrast agents for radiography. The uses of image-based diagnostics are expected to increase substantially. Most imaging techniques use metallic particles or synthetic dyes to visualize the target. Protein nanoparticles, specifically those having inherent fluorescent activity, could be safe, biodegradable and ideal for imaging applications.

### 2.6. Nanocapsules and Core-Shell Zein Nanoparticles

**Figure 10:**
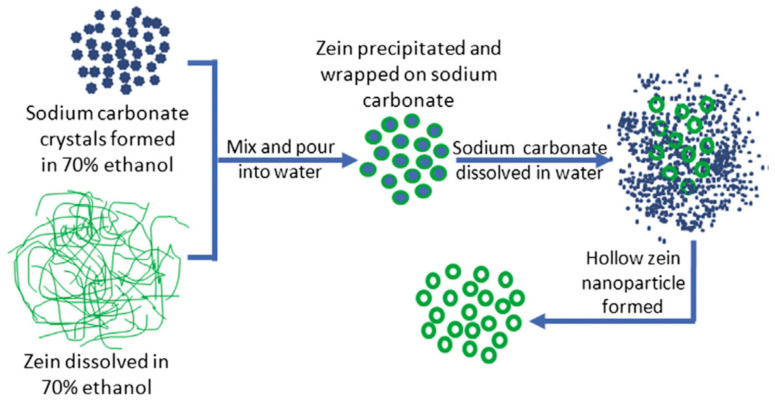
Process of obtaining the hollow zein nanoparticles (nanocapsules). Reprinted with permission from ref. [72]. Copyright 2013 Elsevier.

Nanoparticles are typically spherical in shape and have a solid structure. The loading capability of such solid nanoparticles is low due to the limited surface area available. Hollow nanoparticles, commonly referred to as nanocapsules, have been developed to increase accessibility and facilitate a higher payload capacity. In one such attempt, zein nanoparticles with an average diameter of about 79 nm were developed with a hollow core formation. The hollow core was made possible by using sodium carbonate as sacrificial core templates. The templates were removed after precipitating the particles (Figure 10). The zein nanocapsules were studied for potential drug delivery and the removal of dyes from waste water. Up to 1010 mg of dye per gram of nanocapsules could be sorbed depending on the pH, temperature, and initial dye concentrations used. Being biodegradable and easy to prepare, the zein nanocapsules were considered ideal for treating dye-polluted waste water [71,72]. These nanocapsules were able to load 30% higher metformin compared to solid nanoparticles, and provided a sustained release. The nanocapsules were cytocompatible and capable of entering into the cell cytoplasm, and were detectable using Fluorescein isothiocyanate (FITC) fluorescent dyes [71].

**Figure 11:**
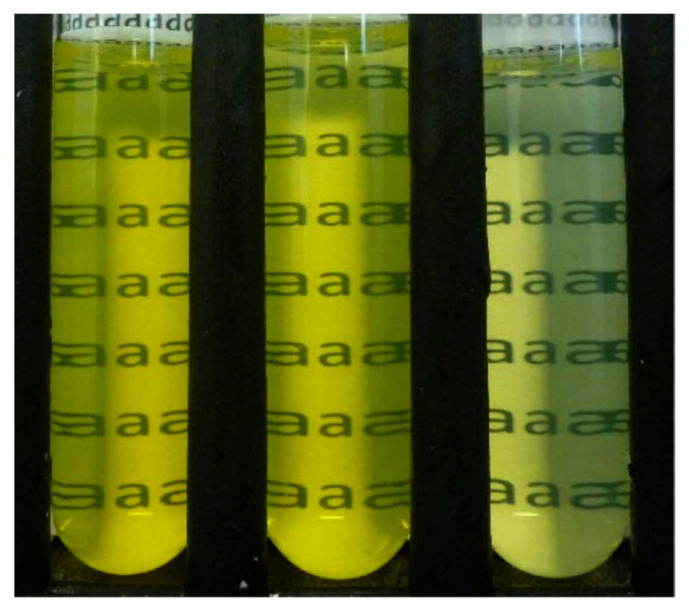
Curcumin-loaded zein-pectin core-shell nanoparticles were able to redisperse in water without any aggregation at three different concentrations of 8.8% (left), 4.25% (middle), and 0.84% (right). Reprinted with permission from ref. [76]. Copyright 2015 Elsevier.

Similar to nanocapsules, core-shell zein nanoparticles have also been prepared using surfactants to improve their stability and performance properties [73]. The precipitation of zein was done at pH 4 to avoid aggregation and low ionic strength. However, the amount of surfactant present (Tween 80) caused variations in the size of the nanoparticles formed. For instance, 1000 nm particles were formed when 0.008% Tween 80 was formed, but the size decreased 10-fold to 100 nm when 0.04% surfactant was present. The addition of the non-ionic surfactant also decreased the charge from +60 mV to +45 mV, and, further, to +26 mV when 0.16% Tween was present. It was found that the size of the zein core was about 76 nm, and the surfactant had formed a shell with a thickness of about 4 nm. The nanoparticles were suggested to have become stable due to the non-ionic surfactant, since the non-polar surfactant decreased attraction between zein, but increased steric repulsion [73]. In another study, zein nanoparticles, used as the core, were coated with alginate and those used as the shell were coated through electrostatic deposition. The core diameter of the nanoparticles was 80 nm and the shell thickness was 40 nm, with a zeta potential of −21 mV. The nanoparticles were observed to have high stability even after being heated at 90 °C for 120 min [74]. Similar results were also observed when core-shell zein nanoparticles were developed using sodium caseinate and sodium alginate. These particles were able to encapsulate zein and increase the water solubility, apparent viscosity, stability to pH, and the salt solution. A substantial improvement in resistance to digestion by anti-gastrointestinal fluids was also observed due to the core-shell structure [75]. In yet another study on developing core-shell nanoparticles, zein was used as the core and pectin, a hydrophobic polysaccharide, was used as the shell, through electrostatic deposition. The diameter of the nanoparticles was about 250 nm, with a narrow PDI of 0.24, and good dispersibility in water was observed. When used to encapsulate curcumin, a high loading efficiency of 86% could be achieved due to the strong hydrophobic interactions. The ability to disperse the curcumin nanoparticles in water without any aggregates (Figure 11) suggested they would be suitable for food and beverage applications and also as dietary supplements [76]. Core-shell zein-lecithin nanoparticles were prepared by the phase separation method for the oral delivery of Rapamycin, a immunosuppressant used during organ transplantation. Vitamin E conjugated polyethylene glycol succinate (TPGS), a non-ionic surfactant, was used to improve the stability of rapamycin in zein-lecithin nanoparticles. The diameter of the nanoparticles formed was 190 nm, the PDI was 0.256 and the zeta potential was −19.7 mV, with high resistance to acids and enzymes in the gastric and intestinal conditions observed. The drug entrapment efficiency and drug loading content were 87% and 26%, respectively. Coating the nanoparticles with TPGS improved uptake by Caco-2 cells and oral absorption, leading to a high bioavailability of 433% compared to free Rapamycin [77]. Zein was used as the core and a hydrophilic anionic polysaccharide (L-carrageenan) was used as the shell, and the structure was crosslinked with cationic calcium ions to form salt bridges. These core-shell nanoparticles were used for the encapsulation and delivery of curcumin and piperine. The core-shell nanoparticles were able to inhibit photo- and thermal degradation, provided sustained release under gastro intestinal conditions, and increased the oral bioavailability of the encapsulated biomolecules [78]. Similarly, zein-pectin core-shell nanoparticles were able to load resveratrol, with good stability between pH 2 and 7 and at 80 °C for 1 h observed. Encapsulation was found to greatly increase the nanoparticles’ antioxidant potential, making them suitable for use in functional foods and as nutraceutical supplements [79].

Hollow or core-shell nanoparticles provide better encapsulation and release properties. However, developing such nanoparticles, particularly on a large scale, may be difficult. Further, controlling the size of the nanoparticles and the extent of their core-shell structures are challenges. Payloads encapsulated inside the hollow structure may have extensive physical and chemical interactions and may not be released easily until the degradation of the nanoparticles occurs. A comprehensive understanding of the properties and behavior of nanocapsules and core-shell nanoparticles from plant proteins is necessary.

**Figure 12:**
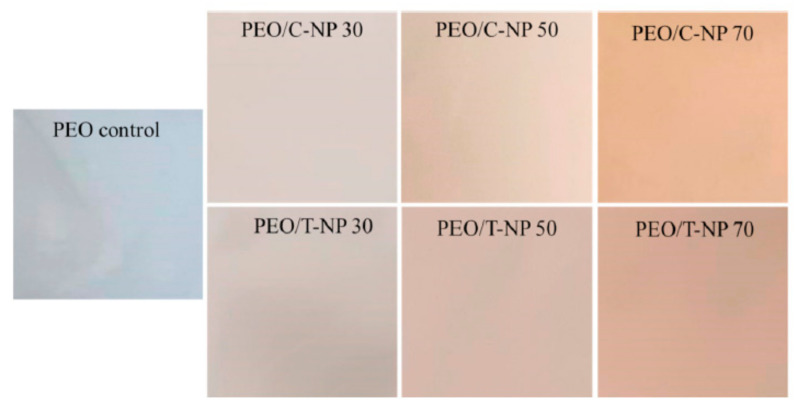
Changes in the appearance and transmittance of polyethylene oxide (PEO) films containing different levels of curcumin-loaded zein nanoparticles. Reprinted with permission from ref. [81]. Copyright 2020 Elsevier 3.

Instead of using chemicals and surfactants, a water soluble polysaccharide extracted from soy proteins was capable of stabilizing zein nanoparticles. The stabilized nanoparticles were 200 nm in diameter, with a PDI of less than 0.2, and did not cause any aggregation between pH 2 and 8, and even at higher temperatures and ionic strengths. When used as a carrier for quercetin, up to 82.5% encapsulation was possible compared to the 59% encapsulation obtained without the soy protein extract coating. In addition to increasing encapsulation, the coating also improved the photochemical stability and scavenging potential, making these nanoparticles suitable for delivering various bioactive molecules for food and pharmaceutical applications [80]. In addition to carrying payloads, zein nanoparticles have also been used to develop films for food packaging. For example, poly(ethylene oxide) (PEO) films were developed by incorporating zein nanoparticles loaded with either thymol or carvacrol. The addition of nanoparticles increased the films’ hydrophobicity and thermal stability. However, the color and opacity of the films increased (Figure 12). Up to 13.6 mg/g of thymol and 15.6 mg/g of carvacrol was released in a sustained manner, and the nanoparticle-containing films were suggested to be suitable for food packaging [81].

## 3. Nanoparticles from Wheat Proteins

### 3.1. Gliadin Nanoparticles

#### Gliadin Nanoparticles for Food Applications

Gliadin is a unique prolamin with glutamine- and proline-rich central parts and terminal areas rich in hydrophobic amino acids, which impart substantial anti-foaming properties. These attributes make gliadin amphiphilic and also provide anti-foaming properties. However, the extent of its antifoaming properties was found to vary with the type of gliadin, with γ-gliadin providing higher stability than α and β gliadins. Similarly, pH affected foaming, since the surface tensions of gliadins were minimum at a neutral pH, higher at an alkaline pH, and highest at an acidic pH. Further, the addition of NaCl improved foaming, whereas positively charged amino acids reduced foaming [82,83]. For instance, gliadin nanoparticles have very poor foam stability and low viscoelasticity at pH 4, but excellent stability and high viscoelasticity at pH 6, although no significant differences are observed in structural or surface properties between the two pH levels. It was suggested that such differences occur during or after adsorption of the nanoparticles due to the formation of films. At pH 6, the covalently crosslinked protein network at the air–water interface was suggested to provide high stability to the nanoparticles [84]. Despite such behavior, gliadins have poor solubility and dispersibility in aqueous solutions, which is a hindrance for biomedical applications. To prepare gliadin with good dispersibility and solubility, gliadin was dissolved in 70% ethanol at 25 °C and precipitated in deionized water to three different nanoparticle concentrations [85]. The particles obtained were spherical in shape with a rough surface, and had an average diameter of 105 nm. The PDI of the particles was 0.078, the surface hydrophobicity was 1711—nearly 9 times higher compared to ovalbumin—and the ζ-potential was +16.2 mV. The extent of foam particles (foamability of up to 174%) and the dimensions of bubbles generated were dependent on the gliadin concentrations. It was suggested that the foaming properties of the gliadin nanoparticles were suitable for food, cosmetic and pharmaceutical applications [85]. A study on the influence of process parameters such as mixing speed and time, and the influence of gliadin concentration on the properties of gliadin nanoparticles formed by antisolvent precipitation, was conducted by Joye et. al. [86]. Nanoparticles with a diameter of about 200 nm were obtained, but these had low stability and thermal resistance, and precipitated quickly. To improve their properties, the nanoparticles were crosslinked with glutaraldehyde, which marginally improved their stability. Hence, it was suggested that glutaraldehyde-crosslinked gliadin nanoparticles did not have desirable properties, and it was necessary to consider alternative approaches to obtain stable nanoparticles for various applications [86].

**Figure 13:**
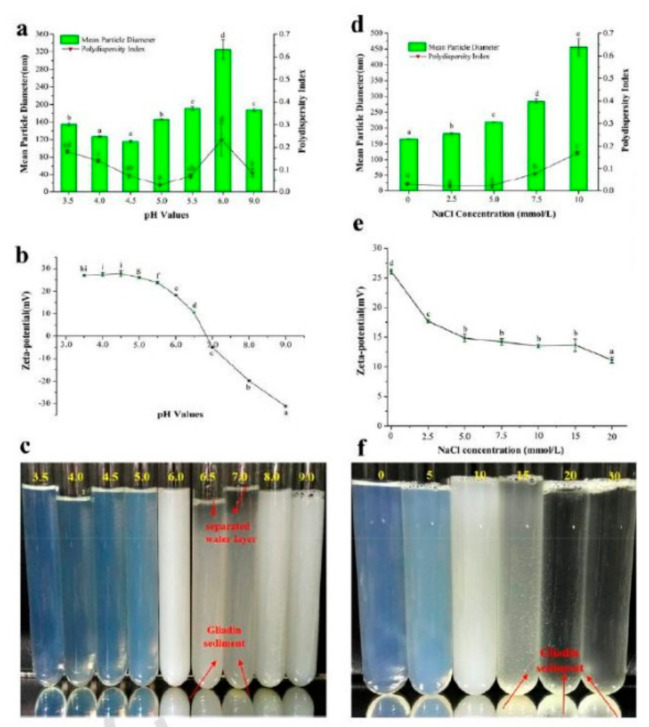
Changes in the size and zeta potential properties of gliadin nanoparticles with different pH (**a**,**b**) and NaCl concentrations (**d**,**e**). Visual images of the gliadin nanoparticles at different pHs (**c**) and NaCl concentrations (**f**). Reprinted with permission from ref. [90]. Copyright 2018 Taylor and Francis.

Coating gliadin nanoparticles with various polysaccharides (octenyl succinic acid (OSA) starch, low methyoxyl pectin (LMP), or high methyoxyl pectin (HMP)), through electrostatic deposition, were considered as possibilities for improving their stability. Coating the nanoparticles with OSA starch caused particle flocculation and precipitation, whereas coating them with the pectins did not cause any flocculation, even at a concentration of 0.10%. Pectin coating almost doubled the particle size and also changed the charge of the nanoparticles from positive to negative, but was found to be beneficial for improving the stability of the particles against pH, ionic strength and thermal treatments [86]. Another approach to increase the stability of the gliadin nanoparticles is through deamidation. The deamidation of gliadin under controlled conditions was also found to substantially alter the stability of the nanoparticles due to increased hydrophobic interactions and hydrogen bonding. The diameter of the deamidated gliadin particles was between 66 and 148 nm at pH 7–9, compared to 379–8332 nm for native gliadin. When used for encapsulating curcumin, an encapsulation efficiency of 91% was possible, with higher thermal stability due to the enhanced hydrogen bonding after deamidation observed [87]. Such enhanced stability was also possible by adding gum arabic during the preparation of the nanoparticles, using the antisolvent precipitation method [88]. Gum arabic formed a coating on the nanoparticles and increased the size of the particles from 190 to 248 at pH 5, and to 270 nm at pH 7. However, the coating provided good stability between pH 4 and 7, and enhanced the ionic strength and thermal stability, even at 80 °C. The improvement in stability was suggested to be due to hydrogen bonding or hydrophobic interactions, depending on the pH. Further, the addition of gliadin nanoparticles (2%) and gum arabic (1 to 4%) into corn oil was found to be useful for producing pickering high internal phase emulsions (HIPEs). Considerable changes to the microstructure, rheology, stability, photodegradation and bioaccessibility of β-carotene was observed when it was encapsulated with the nanoparticles. A three-dimensional network formation was suggested to be responsible for the improved stability. Encapsulation did not affect the lipid digestion or the bioaccessibility of carotenoids [89]. The extent of improvement in the properties of the pickering emulsions due to the addition of gliadin nanoparticles was dependent on the pH, ionic strength, and oil content [90]. For example, the zeta potential of the nanoparticles was −31.2 mV when the pH was 9, compared to +27.8 when the pH was 3.5 (Figure 13). Similarly, the turbidity and zeta potential increased with an increase in pH, due to the changes in the electrostatic interactions.

**Figure 14:**
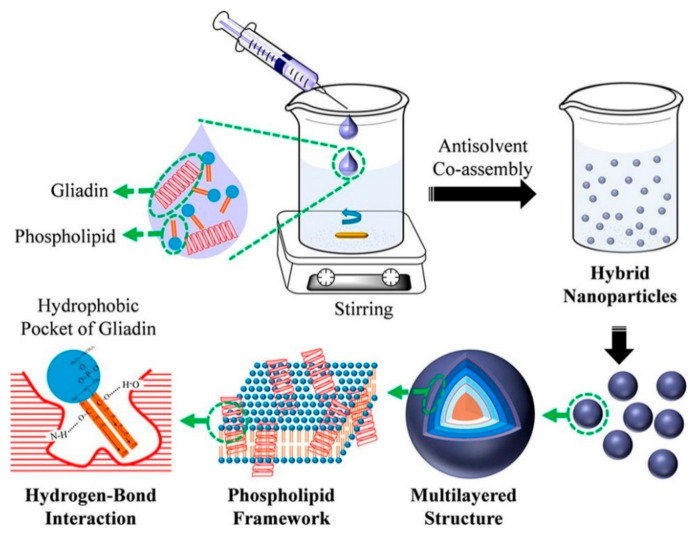
The concept of developing hybrid gliadin phospholipid composite nanoparticles with a gliadin core and phospholipid shells. Reprinted with permission from ref. [91]. Copyright 2019 American Chemical Society.

Unique hybrid gliadin phospholipid nanoparticles were developed with a gliadin-rich core and a phospholipid rich shell using the coassembly and antisolvent coprecipitation approach [91] (Figure 14). Different mass ratios of the proteins and phospholipids were used, and the changes in physical properties were investigated. The particle size increased from 78 to 145 nm when the mass ratio between the gliadin and phospholipids increased from 7:3 to 3:7. Similarly, the zeta potential and PDI were 30.2 and 0.34 for pure gliadin, respectively, but ranged between 16.9 and 23.9 and 0.18 and 0.26 for the hybrid nanoparticles, respectively. The particle size or zeta potential did not show any appreciable change when the nanoparticles were stored for 90 min at pH 4 or 5, even after boiling, or when the salt concentrations varied between 0 and 300 mmol/L. Further, the foamability and foam stability of the hybrid nanoparticles was considerably higher compared to unmodified gliadin nanoparticles, suggesting their suitability for use as emulsifiers and foaming agents [91].

**Figure 15:**
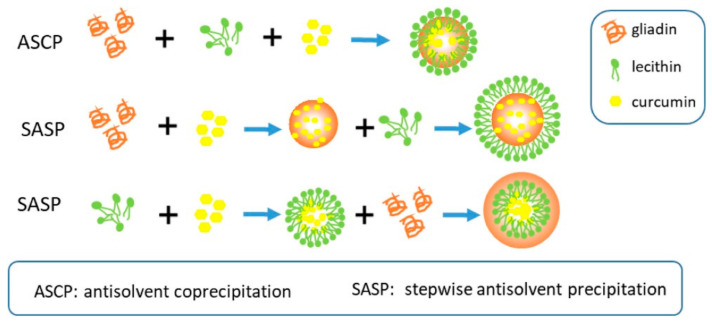
Schematic representation of the ASCP and SASP method of preparing lecithin-containing gliadin nanoparticles for the delivery of curcumin. Reprinted with permission from ref. [92]. Copyright 2018 Elsevier.

Since gliadin is soluble in aqueous ethanol, the most common and convenient approach for preparing nanoparticles is through the anti-solvent coprecipitation (ASCP) process. In a modified process, stepwise antisolvent precipitation (SASP) (Figure 15) of gliadin nanoparticles with lecithin was used to encapsulate and deliver curcumin. The blending sequence influenced the size of the nanoparticles, with the gliadin-lecithin-curcumin nanoparticles having a considerably larger diameter of 264 nm compared to 92 nm for pure gliadin. Curcumin was able to have hydrogen bonding, and electrostatic interactions with gliadin led to reduced hydrophilicity. An encapsulation efficiency of 91% and a loading capacity of 6% was achieved. The nanoparticles were able to protect curcumin against UV irradiation and thermal treatment, and also preserved curcumin’s antioxidant capacity to a higher extent than the conventional ASCP process [92].

Gliadin is suggested to be an ideal protein for drug delivery because it has mucoadhesive properties and adheres to the mucus layer in the stomach, and also has considerably better stability in acidic conditions due to better hydrogen bonding. The nanoparticles obtained through the conventional desolvation process had a diameter of 900 nm with almost no charge, which was considered ideal for encapsulating a sensitive vitamin (α-Tocopherol). A good encapsulation amount of 100 μg of the drug per gram of gliadin, at 77% efficiency, was achievable. However, an initial burst release followed by sustained release occurred through diffusion [93]. In a recent study, gliadin nanoparticles were used as carriers for resveratrol after the nanoparticles were stabilized with gum arabic and chitosan hydrochloride (CHC) [94]. Resveratrol (RES) was added into gliadin dissolved in ethanol in various ratios and the particles were precipitated through the anti-solvent method. Gum arabic and CHC were added into the RES-loaded nanoparticles at different ratios and protein concentrations. The particle size varied between 240 and 908 nm, the PDI was between 0.212 and 0.312, and the zeta potential was between −29 and +12.2 mV. The modified nanoparticles were smaller in size, had better stability, and a high encapsulation efficiency of 68% was possible. The stabilized particles also improved the release of RES and exhibited higher antioxidant and free radical scavenging potential [94]. Similarly, gliadin nanoparticles with diameters of 500 nm were able to load up to 76.4 μg of all-trans-retionic acid (RA) per mg of nanoparticles (efficiency of up to 97%). An initial burst release followed by zero order diffusion release at a rate of 0.065 mg RA/h was observed [95]. Gliadin nanoparticles with an average diameter of about 900 nm showed entrapment efficiencies between 52 and 82%, and drug concentrations between 550 and 980 μg/g of gliadin when used to load drugs with different polarities. It was found that stronger interactions occurred between gliadin and apolar drugs compared to cationic and amphiphilic drugs. Such interactions provided a low diffusion coefficient and partition coefficient and hence low permeability of the gliadin, which are preferable for controlled drug delivery [96].

Gliadin nanoparticles were prepared using the electrospray technique for their potential use as drug carriers for the delivery of anticancer drugs. For the electrospray technique, gliadin (7%) was dissolved in 70% ethanol at room temperature for 2 h and aged for 2 days. The solution was placed in a syringe and electrospraying was carried out using a syringe pump at a flow rate of 0.5 mL/h and voltages between 12 and 14 kV. In addition to pure zein, composite nanoparticles of zein and gelatin were also prepared and later crosslinked with glutaraldehyde. The potential of the nanoparticles to load and release cyclophosphamide, an anticancer drug, was studied [97]. Some of the properties of the nanoparticles obtained are given in Table 4. The gliadin nanoparticles did not cause any cytotoxicity, but those loaded with the anticancer drug resulted in the considerable apoptosis of MCF7 breast cancer cells [97].

Although gliadin has several desirable features for drug delivery and other medical applications, preparing stable gliadin nanoparticles is a challenge. Further, there have been contradicting results on the cytotoxicity of gliadin proteins. The extraction of gliadin from wheat gluten is tedious and also makes the proteins expensive. Gliadin-free gluten will be rich in glutenin and starch, and may not find suitable applications. Hence, the large-scale production of gliadin nanoparticles could face several technical and economic hurdles. Nevertheless, further studies are necessary to develop stable nanoparticles and to clearly understand the reasons for the cytotoxicity of gliadin.

### 3.2. Nanoparticles from Wheat Glutenin

**Figure 16:**
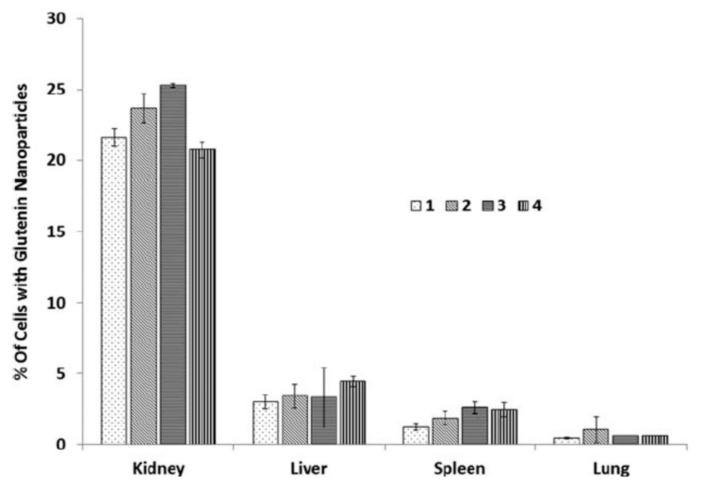
Biodistribution of wheat glutenin nanoparticles in different organs in mice. Reprinted with permission from ref. [98]. Copyright 2015 John Wiley and Sons.

**Figure 17:**
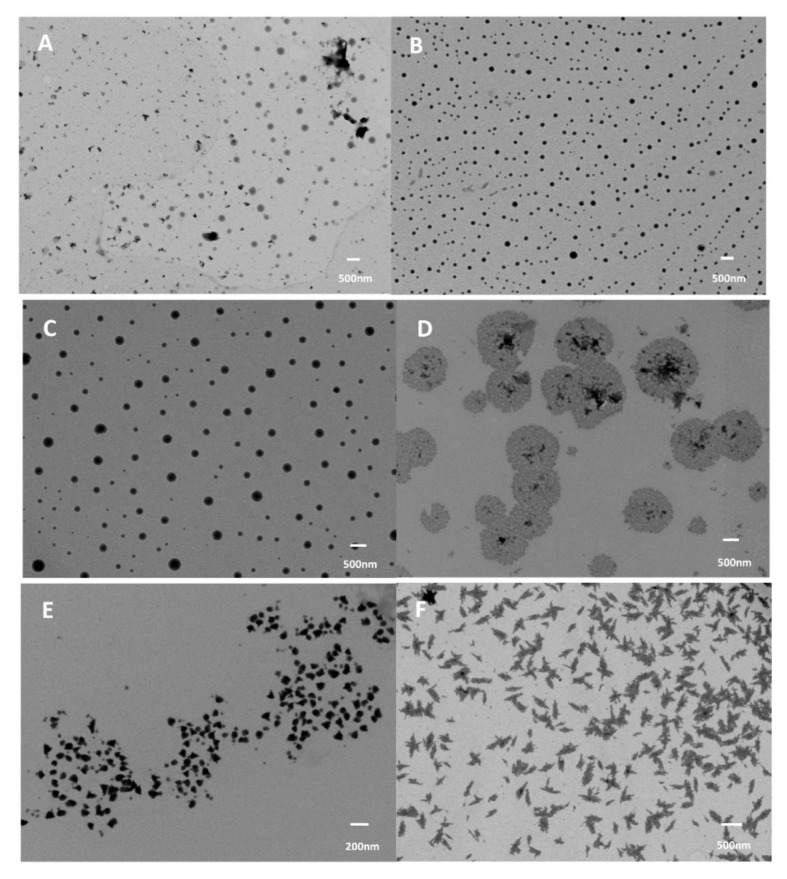
Differences in the morphology of wheat glutenin nanoparticles prepared at different oxidation times of 10 h (**A**), 20 h (**B**), 30 h (**C**), 40 h (**D**), 50 h (**E**), and 60 h (**F**). Reprinted with permission from ref. [99]. Copyright 2019 Elsevier.

Glutenin is the high molecular weight protein in gluten and, unlike gliadin, does not dissolve in aqueous ethanol or other solvents. To prepare nanoparticles, wheat glutenin extracted from wheat gluten was treated with sodium bisulfite in a 20:1 ratio for 4 h at 70 °C. Later, hydrolyzed glutenin proteins were obtained by treating the wheat glutenin with 0.1 M sodium hydroxide for 2 h at 80 °C. These hydrolyzed proteins were dissolved in ethylene glycol, to which water with different pHs was added to form the nanoparticles by phase separation. The nanoparticles obtained had diameters between 70 and 140 nm, with smaller particles forming at high acidic and alkaline pHs, and the zeta potential of the nanoparticles varied between −200 and +320 mV. The particles were cytocompatible and were found to be capable of entering various organs in mice, predominantly the kidneys, as seen in Figure 16 [98]. In a study by Li et al., [99] redox sensitive wheat glutenin nanoparticles were prepared for food, medical, and other applications. Glutenin was dispersed in acetic acid in various ratios, to which β-mercaptoethanol was added to assist in the reduction of disulfide bonds. The degraded glutenin was separated, and insoluble proteins were collected which were redispersed in water. Nanoparticles were formed through the co-solvent approach, using hydrogen peroxide as the oxidizing agent, with the oxidation time varying from 10 to 60 h. The potential of loading and releasing Nile blue, a hydrophobic compound from the nanoparticles, was also studied. The size (100–300 nm) and morphology of the nanoparticles varied substantially depending on the oxidation time (Figure 17). Up to 78% encapsulation efficiency of Nile blue A was possible but no in vivo or in vitro release studies were performed [99]. Dry red-ox sensitive particles were obtained by freeze-drying [99]. The particles formed had irregular morphology, and their size ranged between 50 and 200 nm. Oxidation substantially changed the shape and size of the nanoparticles depending on the protein and peroxide concentration and the stirring time. Oxidation increased intramolecular interaction and reduced the particle size to as low as 12 nm. A glutenin concentration of 0.83% and an oxidation time of 20 h was considered optimum and produced nanoparticles in the range of 100–300 nm [99]. Although studies on glutenin have been limited, it has been demonstrated that glutenin is non-cytotoxic and suitable for medical applications. However, preparing nanoparticles from glutenin is difficult, since it does not dissolve in common solvents. The in vivo degradation of glutenin is also unknown, and glutenin nanoparticles may not easy dissolve under physiological conditions, limiting their use for controlled release applications.

## 4. Nanoparticles from Soy Proteins

### 4.1. Soy Protein Nanoparticles for Controlled Release Applications

Soy proteins are one of the most pure and commonly available proteins extensively used for food and non-food applications. Although soy proteins do not dissolve in common solvents, several approaches have been used to prepare nanoparticles. Conventionally, soy protein nanoparticles are prepared through either ethanol desolvation, Ca^2+^-induced cold gelation, or a combination of the two processes. For instance, a process involving dispersion, desolvation, drug incorporation, crosslinking, and evaporation was followed to develop soy protein nanoparticles as carriers for curcumin [100]. In this approach, soy protein isolate was dispersed in deionized water and later ethanol, as a desolvating agent, was added dropwise, varying the concentration of the protein and ethanol. Glutaraldehyde was added into the solution and the crosslinking was done for 16 h at room temperature. Later, the ethanol was removed and the nanoparticles were obtained by lyophilization. A similar procedure was used to load curcumin onto the nanoparticles. Curcumin-loaded soy protein nanoparticles had an average diameter between 220 and 287 nm, and a zeta potential around −36 mV depending on the ratio of soy protein to curcumin (Table 5). The highest encapsulation efficiency of curcumin was 97%, and the loading efficiency was 2.7%. Up to 80% curcumin was released in PBS within 8 h in a relatively sustained manner, indicating these nanoparticles’ suitability for controlled release applications [100].

Composite nanoparticles were developed from soy proteins and cellulose nanocrystals (CNCs) as potential delivery agents for curcumin. Soy proteins were dispersed in water, the pH was adjusted, and CNCs were dispersed in water at a concentration of 0.25 mg/mL, whereas curcumin was dissolved in anhydrous ethanol at concentrations between 0.2 and 0.5 mg/mL. To prepare the nanoparticles, curcumin solution was added into soy protein solution and stirred at 400 rpm for 2 h. Later, the CNC solution was added dropwise and mixed for 1 h. The composite nanoparticles formed were collected by centrifugation and the evaporation of the solvent. The size of the particles and the zeta potential varied depending on the ratio of soy protein and CNC used, with 6:1 being the most optimum concentration. At this ratio, a nanoparticle composite with an average diameter of 198 nm and a PDI of 0.14 was obtained. A high curcumin encapsulation efficiency of 88% was possible. The release of curcumin was considerably low in the stomach and hence improved targeted delivery could be achieved [101]. Soy protein nanoparticles with diameters of 50–52 nm were prepared after subjecting them to high intensity ultrasonication. This ultrasonication increased the uptake of curcumin to 144.5 μg/mg, compared to 103.9 μg/mg before the modification. The higher loading ability (95.7 μg/mg against 64.2 μg/mg) was retained even after the nanoparticles were freeze-dried and reconstituted. The curcumin encapsulated in the nanoparticles had improved storage stability due to the increased hydrophobicity of the proteins after ultrasonication [102]. Generally, nanoparticles are used as carriers for curcumin. In an alternative approach, curcumin was made into nanoparticles and coated with soy and other proteins to increase stability and bioavailability. Aqueous dispersions of curcumin nanoparticles were stabilized, the loading capacities were enriched with sodium caseinate, and whey protein isolate coating was done, leading to a higher loading capacity of 27 and 21%, respectively, compared to 12% for the soy protein-coated biomolecules [103]. However, soy protein-coated particles had higher thermal stability compared to curcumin particles coated with other proteins.

Soy protein nanoparticles have also been conjugated with various biomolecules for specific applications. For example, soy protein isolate was conjugated with folic acid and made into nanoparticles for the delivery of curcumin as a model drug. To prepare the nanoparticles, the untreated or conjugated nanoparticles were dissolved in water (15–30 mg/mL) at pH 7.5–8.0. Ethanol was added dropwise into the soy protein dispersion to precipitate the nanoparticles. A similar procedure was followed to load curcumin after dissolving curcumin in ethanol. Glutaraldehyde was used to crosslink the nanoparticles and improve stability [100,104]. The size of the nanoparticles was between 150 and 170 nm, and the zeta potential was between −35 to −42 mV depending on the degree of conjugation. The conjugation provided higher yield and better nanoparticles. A curcumin encapsulation of up to 92.7% was possible for the conjugated particles, but a faster release rate was observed. Caco-2 cells had a higher uptake of the modified nanoparticles without causing any cytotoxicity, suggesting their suitability for targeted drug delivery [104]. In another study, soy protein nanoparticles were prepared through alkaline hydrolysis and crosslinking with glutaraldehyde. These nanoparticles were conjugated with a snake antivenom (IgG) using 1-ethyl-3-[3-dimethylaminopropyl] carbodiimide hydrochloride (EDC) as the conjugation agent, and vortexing for 10 min at 25 °C [105] was done. At the optimum condition of treating soy proteins with 5.4% alkali and 28 μg/mg of glutaraldehyde, nanoparticles with an average size of 71 nm and a zeta potential of −28 mv were obtained. After conjugation of the antivenom, the size of the nanoparticles increased to 600 nm, but there was considerably higher inhibition of the protease, phospholipase, and hyaluronidase enzymes produced by *Bungarus caeruleus* venom by the encapsulated antivenom compared to free antivenom [105].

Soy proteins have been modified with enzymes to obtain water soluble components and to increase payload capabilities. In one such attempt, a low molecular weight (18 to 80 kDa) soy protein was obtained after it was treated with compound enzymes. The hydrolyzed soy protein was dialyzed to obtain water soluble proteins with molecular weights of 8 kDa, which were made into nanoparticles using the desolvation method. The nanoparticles were crosslinked with EDC (1-ethyl-3-(3-dimethylaminopropyl)carbodiimide/N-hydroxysuccinimide (EDC/NHS) and conjugated with folic acid in the presence of EDC/NHS, and the modified nanoparticles were used for the loading of doxorubicin for controlled delivery to tumors. The diameter of the nanoparticles was 207 nm, but increased to 232 nm after the conjugation (grafting) of folic acid. Similarly, the zeta potential decreased from −20 to −31 mV for unconjugated and from −28 to −42 mV for the conjugated particles when the pH was increased from 5 to 10. A doxorubicin encapsulation efficiency of 96.7% and loading efficiency of 23% was possible. Folic acid-modified nanoparticles provided considerable better tumor penetration and accumulation and hence much higher activity against tumors compared to unmodified soy protein nanoparticles [106]. In another study, soy protein isolates treated with glutaminase caused changes in the secondary structure, leading to greater protein unfolding. Modified soy proteins were made into nanoparticles and used as potential carriers for curcumin. Some of the properties of the nanoparticles before and after modification are given in Table 6. Substantial improvements in encapsulation efficiency, loading amounts, and stability were observed, making the nanoparticles suitable for the delivery of curcumin [107]. The enzymatic hydrolysis of soy proteins to different extents was considered suitable for developing nanoparticles for food applications. Enzymatic hydrolysis was done by treating the protein dispersion with Flavorzyme at pH 7, 50 °C and Alcalase (pH 8, 55 °C) or protamex (pH 7, 50 °C) to obtain hydrolysates with a degree of hydrolysis of 3, 7 and 11%, respectively [108]. The nanoparticles obtained were spherical in shape, with diameters between 80 and 170 nm. Hydrolysis changed the secondary structure from α-helix to β-sheet, which impeded nanoparticle formation. However, the particles formed from hydrolyzed proteins had good emulsifying properties, improved surface hydrophobicity, and antioxidant properties.

Folic acid-modified soy protein nanoparticles were also prepared for the delivery of the drug doxorubicin, and the cellular uptake and cytotoxicity were evaluated [109]. To prepare nanoparticles, soy protein isolate was treated with enzymes to hydrolyze the proteins and reduce their molecular weights. Later, the soy proteins were modified with folic acid through the interaction between the carboxylic groups in folic acid and amino groups in soy proteins. A biopolymer-monomer polymerization process was used to obtain nanoparticles without the need for any organic solution, surfactants or crosslinkers. In this process, soy protein and N-3-Acrylamidophenylboronic acid were dispersed in water and heated to 90 °C under nitrogen atmosphere. The solution was dialyzed using a 14 kDa membrane and the nanoparticles were collected. The zeta potential of the nanoparticles (200 nm diameter) was between −15 mV and −32 mV, depending on the pH. Modifying the nanoparticles with folic acid not only improved stability but also increased the accumulation and penetration of the nanoparticles into SH-SY5Y cells. Doxorubicin-loaded nanoparticles had a long circulating time and were able to accumulate in tumors to a larger extent than free drug or unconjugated soy protein-drug particles. A substantial decrease in the size of tumors in mice was noticed when the drug-loaded nanoparticles were injected into mice with H22 tumors [109]. The ability of soy protein nanoparticles to load β-carotene and assist its release in the digestive system was studied by Yi, et al. [108]. In this study, soy protein isolate was dispersed in water, to which 0.1% beta-carotene dissolved in ethyl acetate was added in a 1:9 ratio. Extensive homogenization was conducted at 70 MPa. Ethyl acetate was removed and the protein particles were lyophilized to obtain a beta-carotene content of 10.7 mg/g of soy protein. The average diameter of the nanoparticles was 372 nm. The nanoparticles were able to release 74 and 60% beta-carotene in gastric and intestinal juices, respectively [110].

Soy protein nanoparticles can also be formed through cold gelation, where the proteins are first hydrated in water and then treated in pH 12 solution at 85 °C for 30 min. Later, the pH was reduced to 7, 8, or 9, and CaCl_2_ was added in different molar ratios to form the particles after incubation overnight at room temperature. Nanoparticles with sizes between 28 and 179 nm were obtained, depending on the calcium concentration and pH (Table 7). Typically, higher levels of calcium and a lower pH formed larger particles with a lower surface charge and hydrophobicity. The nanoparticles did not show any cytotoxicity to Caco2 cells and were able to enter the cytoplasm [111]. In another study, soy protein nanoparticles with average diameters of 30, 99, and 181 nm were prepared as carriers for vitamin B_12_. The PDI of the particles varied between 0.27 and 0.31, and the zeta potential was within a narrow range of 0.021 to 0.030. However, the loading capacity and loading efficiency for B_12_ were considerably low, and varied between 0.021 and 0.030 and 10.3 and 13.5% [112]. The nanoparticles were not only cytocompatible to Caco-2 cells but were able to internalize into the cytoplasm. Up to 2 to 3 times higher delivery of B_12_ was possible depending on the size, suggesting the suitability of the nanoparticles for the oral delivery of vitamins.

**Figure 18:**
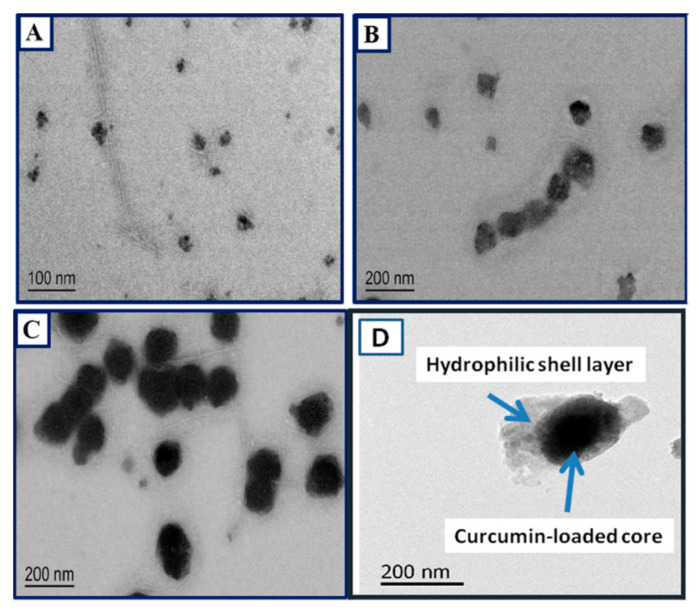
TEM images of untreated (**A**) and re-assembled β-CG before (**B**) and after loading curcumin (**C**,**D**) shows the core-shell formation of the nanoparticles. Reprinted with permission from ref. [114]. Copyright 2019 American Chemical Society.

Soy protein nanoparticles can also be formed in the core-shell form for the delivery of different biomolecules. In one such attempt, nanoparticles (100–500 nm diameter) formed from soy protein isolates and loaded with curcumin were coated with a soy polysaccharide to form a core-shell complex due to their varying complexation properties with pH. The size and properties of the core-shell structures were dependent on the pH, with acidic pH (4.0) providing better thermal stability and control release properties. The bioaccessibility of curcumin was not compromised by the core-shell structure or the coating. Nanoparticles showed good redispersion, and those prepared at pH 4 had sustained release of 56–60% within 24 h [113]. In another alternative approach to develop core-shell nanoparticles, β-conglycinin (β-CG), a storage globulin in soy proteins, was made into nanoparticles for the delivery of curcumin. A unique process of disassembly and reassembly in the presence of urea was used to develop the core-shell structure. When urea concentrations higher than 4 M were used, the β-CG denatures into several sub-units. These sub-units reassembled into a core-shell structure when urea was removed through dialysis. Curcumin crystals were added into the β-CG solution when high urea was present. During the disassociation, curcumin preferentially aligned with the β subunits and were hence encapsulated in the core (Figure 18). Up to 18 g of curcumin per 100 g of protein could be loaded with high solubility, thermal stability, increased bioavailability and sustained release [114]. A similar phenomenon and the formation of core-shell β-CG nanoparticles was possible when urea was replaced with high concentrations (30%) of ethanol. The size and shape of the nanoparticles formed could be controlled by varying the concentration of ethanol. A maximum curcumin loading of 13.7 g per 100 g protein was possible, with considerably higher stability and bioavailability compared to pure curcumin observed [115].

**Figure 19:**
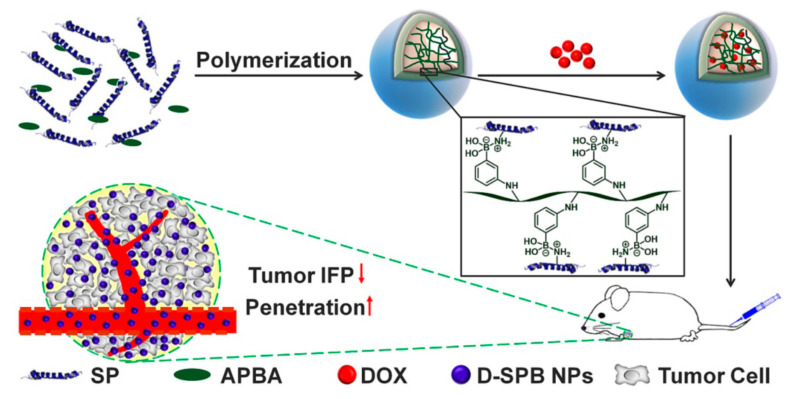
Representation of preparing and delivering soy protein nanoparticles into tumor cells. Soyprotein (SP), acrylamidophenylboronic acid (APBA), Doxorubicin (DOX), phenylboronic acid-decorated soy protein nanoparticles (D-SPB) Reprinted with permission from ref. [116]. Copyright 2019 Ivyspring International Publisher.

Soy protein, particularly, the β-CG component was considered to decrease tumor interstitial fluid pressure and could actively target and improve the tumor microenvironment for enhanced cancer therapy. To prove this hypothesis, soy proteins were purified to obtain a water soluble component with a molecular weight of 35 kDa. Nanoparticles were prepared based on the polymer—monomer pair reaction system and decorated with phenylboronic acid, as represented in Figure 19. Three separate groups of nanoparticles with average diameters of 30, 50, and 100 nm, respectively, were prepared by varying the pH, and were used as carriers for doxorubicin, a model anticancer drug. Nanoparticles obtained under all the conditions were spherical in shape and showed excellent stability in water, PBS, Dulbecco’s Modified Eagle Medium (DMEM), and 10% fetal bovine serum (FBS) even after incubation for 96 h. The PDI of the three nanoparticles was between 0.14 and 0.21, and the zeta potential was between −12 and −23 mV. Although all sizes of the nanoparticles were able to internalize in cells, the smaller diameter particles showed a higher penetration. The modified nanoparticles showed an affinity for sialic acid in tumors and were found to decrease tumor interstitial fluid pressure and solid stress. Pre-treatment and the ability to obtain smaller diameter (30 nm) nanoparticles enabled the accumulation of the anti-cancer drug and higher anti-tumor efficacy [116]. Similar to β-CG, a component in liphophilic protein (LP) was extracted and combined with soy proteins and made into nanoparticles with a diameter of 170 nm. The protein complex had a higher loading capacity (26%), and provided resistance to oxidation and a sustained release of linoleic acid, which was conjugated to the proteins. Further, coating the nanoparticles with sodium caseinate improved the colloidal stability and sustained release in a simulated gastrointestinal tract [117]. Soy proteins are more readily available than zein or gliadin and hence could be preferred for the preparation of protein nanoparticles. However, unlike zein or gliadin, soy proteins do not dissolve in alcohols or common solvents and it is therefore difficult to make them into nanoparticles. The extraction of soluble components, blending, and chemical modifications, as discussed above, are necessary to obtain soy protein nanoparticles for commercial applications.

### 4.2. Applications of Soy Protein Nanoparticles in the Food Industry

**Figure 20:**
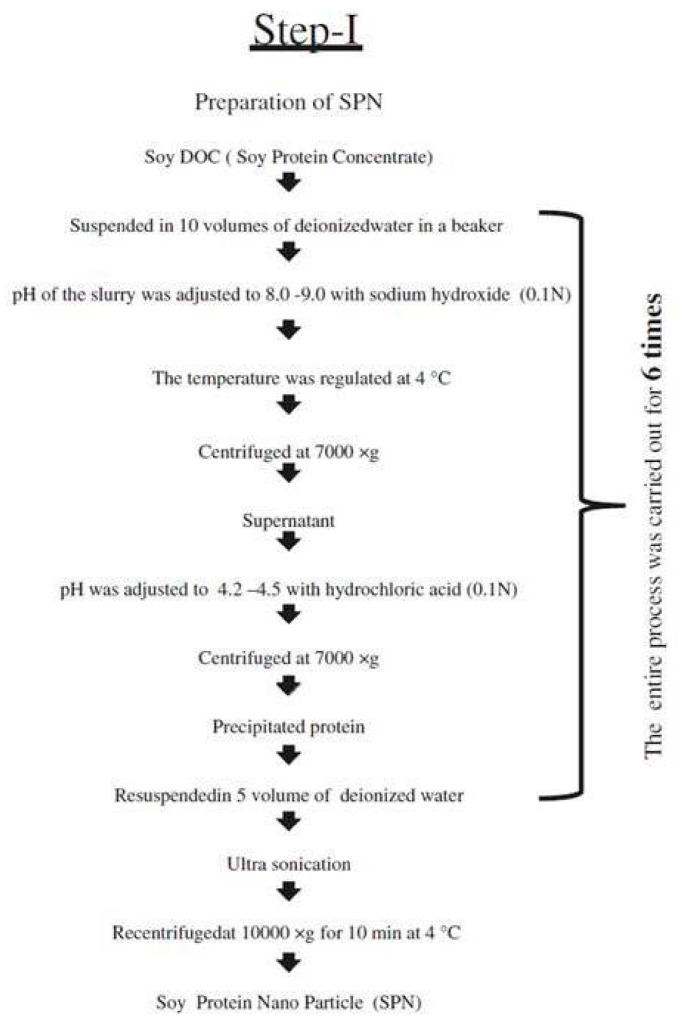
Steps used for the preparation of soy protein nanoparticles to achieve a diameter of 72 nm. Reprinted with permission from ref. [123]. Copyright 2019 John Wiley and Sons.

Various approaches have been used to prepare soy protein nanoparticles for stabilizing pickering emulsions [118]. Soy protein solution (6%) was prepared by dispersing in distilled water and hydrating at 4 °C overnight. The protein solution was heated at 95 °C for 15 min and different concentrations of NaCl were added into the solution to change the ionic strength and induce the formation of nanoparticles. These nanoparticles (about 100 nm in diameter) were added into pickering emulsions to improve their stability and storage properties [118]. The emulsions showed substantial improvement in coalescence and creaming stability after the addition of the nanoparticles. Using a simple heat-induced soy protein nanoparticle aggregate for stabilizing food products was considered to be unique and to possess considerable practical importance [118]. Another method to increase the stability of Pickering emulsions is to use soy protein nanoparticles formed through Ca^2+^ aggregation and crosslinking with glutaraldehyde, as seen in another study. The properties of the nanoparticles were dependent on the concentration of Ca^2+^, with increasing concentrations leading to increases in particle sizes from 60 to 130 nm. Crosslinking also increased particle size, surface charge, and emulsion stability, but decreased hydrophobicity. It was reported that the surface coverage of 3.8–12.6% provided by the soy protein nanoparticles was considerably lower than many pickering emulsions reported earlier, and they are hence suitable for use in functional foods and medicine [119]. Further, it has been reported that the addition of sodium chloride (100–500 mM) to the soy protein nanoparticle-stabilized pickering emulsions substantially altered the particle size, surface hydrophobicity, and zeta potential. NaCl also increased the freeze-thaw stability, the extent depending on when the salt was added and the ionic strength applied. Adding the salt before emulsification led to the formation of a gel-like network beneficial for creaming [120]. Pickering emulsions with substantially improved emulsion and oxidative stability and with resistance to in vitro digestion were obtained when soy protein and anthocyanins were made into complex nanoparticles [121]. Food grade emulsions and emulsion gels have also been prepared with the aid of soy protein nanoparticles. In one such attempt, soy protein nanoparticles and a pectin complex were made, and glycyrrhizic acid nanofibrils were added [122]. Lower nanofibril concentrations in the nanoparticle complex reduced interfacial tension and provided a lower droplet size, whereas the flocculation and clustering of oil droplets was observed at higher (1–2%) concentrations. Self-standing emulsions gels with good gel strength, shear sensitivity, and thixotropic recovery were obtained, due to the addition of the nanoparticle complex [122]. Nanoparticles with a size of 72 nm, reduced from the initial size of 587 nm, could be obtained after repeating the procedure (Figure 20) six times. The addition of nanoparticles to yogurt increased its antiradical scavenging activity, ferric reducing-antioxidant properties, and considerably higher oxidative stability and proteolytic activity could be obtained, emphasizing the application of soy protein nanoparticles for developing functional food products [123].

Soy protein emulsions prepared using nanoparticles produced in a microfluidizer were studied for changes in particle size, morphology and rheology. For the preparation of the nanoparticles, soy protein isolates were dispersed in water and intensively homogenized, and the pH was adjusted to various levels. This dispersion was fed to a microfluidizer operating at 159 MPa for several cycles. A rapid change in aggregate size from 6.5 to 0.5 μm occurred after certain cycles. The aggregates contained nanoparticles with diameters from 69 to 132 nm depending on the pH (7 or 9) and microfluidizing concentrations (4, 7, or 10%) [124]. The viscosity and storage loss modulus decreased as the soy protein aggregates were passed through the microfluidizer, but only marginal changes in the fractal dimensions was observed [124]. A liphophilic protein containing hydrophobic proteins and phospholipids was extracted from soy proteins and used to develop nanoparticles for stabilizing oil in water emulsions. Nanoparticles with a diameter of 136 nm, a zeta potential of −20 mV and improved dispersibility were obtained. Oil in water emulsions stabilized by the liphophilic protein nanoparticles, when combined with a detergent (1–4% of Tween 20), provided better physical stability in the presence of NaCl, and after heating to 90 °C, compared to the stability provided by the β-conglycinin and glycinin components of soy proteins. Such an improvement was suggested to be due to the formation of a rough granular film at the air–water interface and a synergistic effect between the hydrophobic proteins and phospholipids [125]. In addition to the extracted components, self-assembled soypeptide nanoparticles were formed using ultrasound and used as a stabilizer for oil-in-water emulsions. The particles obtained had a size of 104 nm and a PDI of 0.2 [126]. The nanoparticles were able to cover the oil–water interface and hence prevented coalescence, lipid oxidation, and the formation of hexanol during storage. Adding a surfactant formed a mixed interfacial layer, increasing the emulsifying ability. The nanoparticles acted as bifunctional emulsifiers and could be used to prepare stable oil–water emulsions without any loss of soy protein due to hydrolysis [126].

Improving the functional and nutritional properties of food products by using nanoparticles is feasible and desirable. Soy protein nanoparticles offer unique benefits for food applications both in terms of their properties and their scale of production and cost. However, most food applications of soy protein nanoparticles have been conducted at the laboratory stage. Further studies are necessary to understand the behavior of soy protein nanoparticles when used on a commercial scale and for common food applications.

## 5. Nanoparticles from Miscellaneous Plant Proteins

In addition to the proteins from major food crops discussed above, most plants contain proteins. Researchers have extracted, characterized, and utilized proteins from various plant sources for the preparation of materials, including nanoparticles. In one such attempt, proteins from the medicinal plant *Radix Pseudostellariae* were extracted and later dispersed in phosphate buffer solution and heated to 100 °C for 30 min. Self-assembled nanoparticles precipitated when the protein solution pH was adjusted to 5.7. The size of the nanoparticles was between 35 and 150 nm and the PDI was 0.2 when the pH was between 5.6 and 5.9. The curcumin loaded onto the nanoparticles showed the high thermal and light stability required for food and pharmaceutical applications [127].

Proteins in barley contain 45% hordein and 40–45% glutelin, which are known for their good foaming and emulsifying properties. Hence, barley proteins have been used to develop both micro- and nanoparticles [128,129]. A unique pre-emulsification and microfluidizing method was used to prepare the nanoparticles without the need for any organic solvents or crosslinking agents [126]. In this process, aqueous protein suspension was combined with canola oil containing 0.05% β-carotene in a 1:1 ratio. This emulsion was passed through a microfluidizer system at 350 bar, where the particles were formed and later spray-dried to obtain powdered nanoparticles [128]. The processing pressure, recirculation times, protein concentration, and oil-to-protein ratios were varied to obtain nanoparticles with different properties. Both the particle size and the PDI varied significantly with the pressure and number of recirculations. The nanoparticles had a high zeta potential of −35 mv, good storage stability, and were able to load 51–54% of lipophilic nutraceutical compounds. These nanoparticles were degraded by pancreatin, did not show any cytotoxicity to Caco-2 cells, and were found to accumulate in the cytoplasm. The properties of the nanoparticles and the encapsulation efficiency of the lipophilic compounds were dependent on the processing conditions [129].

Nanoparticles produced from pea protein isolates were crosslinked using calcium ions for the protection and delivery of reseveratrol. Properties of the nanoparticles were dependent on the pH and calcium ion concentration used (Table 8). The formation of salt-bridges, hydrophobic interaction, and hydrogen bonding were suggested to form between the proteins and calcium ions. A drug loading of 30 μg/mg of protein, and an encapsulation efficiency of 74% was possible, mainly due to hydrophobic interactions. In addition to physiochemical stability, the antioxidant properties of the drug were also enhanced, making them ideal for delivering nutraceuticals for the food and pharmaceutical industries [130].

Walnut protein isolates were made into nanoparticles using the electrospraying approach, where the protein solution was prepared in water and later diluted with 2-propanol. The aqueous alcohol solution was used to electrospray at voltages between 7.5 and 22.5 kV, and a solution flow rate of 0.06–0.2 mL/h. A similar procedure was also used to prepare curcumin-loaded nanoparticles. The conditions used for preparing the nanoparticles and their properties are given in Table 9. The particles formed were spherical in shape and had an average diameter of 142 nm before, but increased to 297 nm after loading curcumin (Table 9). An encapsulation efficiency of 61.5% of curcumin was possible. In vitro studies showed that most of the curcumin was released in the small intestine and to a limited extent in the stomach. Considerably high antioxidant activity was observed after digestion of the nanoparticles due to the release of bioactive peptides, suggesting the suitability of the NPs for food applications [131]. In another study, proteins were extracted from sunflower seeds after treating the seeds in 10% NaCl at pH 6 for 60 min and at 37 °C. The filtrate obtained was precipitated and washed with HCl to obtain the proteins. Nanoparticles were obtained by redissolving the proteins in 10 mM NaCl and adding ethanol dropwise into the solution. Curcumin dispersed in methanol and 8% glutaraldehyde were also added to crosslink and stabilize the nanoparticles [125]. The average size of the nanoparticles obtained was 197 nm. A curcumin encapsulation efficiency of 83% was achieved with good stability in water and gastrointestinal conditions. Curcumin-loaded nanoparticles had good antioxidant and anti-inflammatory effects, with an IC_50_ of 45.3 μM for the inhibition of lipoxygenase [132].

Nanoparticles were developed from peanut proteins by the addition of CaCl_2_. Hydrated peanut protein powder was subject to a four step process in which the first step involved treating the protein powder in 0.1 M NaOH. In the second step, the suspensions were maintained at 85 °C for 30 min and later cooled to room temperature, and the pH was adjusted to 7 (step three). In the final step, CaCl_2_ with concentrations ranging from 2.5 to 7.5 mM were added to initiate nanoparticle formation after being stored overnight at room temperature [133]. Nanoparticles had diameters between 80 and 140 nm, with a PDI between 0.178 to 0.42 depending on the pH, calcium ion concentration, and type of drying used. The nanoparticles retained excellent thermal stability and were able to resist any change due to the pH in the gastrointestinal track. No major change in the morphology of the particles was observed, even after storing for 60 days at room temperature, suggesting the suitability of these nanoparticles for the delivery of various bioactive compounds [134]. Peanut proteins were isolated from peanuts with a protein purity of 92%. Peanut protein nanoparticles were formed by a thermal treatment of 8% solution and the addition of NaCl to obtain the desired ionic strength. The proteins were dispersed in distilled water and left overnight at 4 °C. Later, the pH was adjusted to 7 and the solution was heated at 100°C for 20 min, cooled, and the particles obtained were collected. The size (178 to 261 nm), zeta potential (−36 to −8 mV) and PDI (0.47 to 0.25) of the nanoparticles varied depending on the ionic strength of the solution. The addition of the nanoparticles to pickering emulsions decreased the droplet size but improved coalescence and creaming stability [135]. Using a similar approach, selenium-enriched peanut protein nanoparticles were made into pickering emulsions and later used to encapsulate a unique polymethyoxyflavone (5-demethylnobiletin (5DN)) to treat cancer. Encapsulation in the nanoparticles increased the bioaccessibility, cellular uptake, and the rate of transport of the drug. Due to the emulsion system, the protein nanoparticles could be digested in the GI tract and release 5DN. The mixed micelles also enabled absorption of the drug by Caco-2 monolayers. Hence, developing a nanoparticle-based pickering emulsion system was suggested to be suitable for increasing bioavailability and for targeted delivery [135].

**Figure 21:**
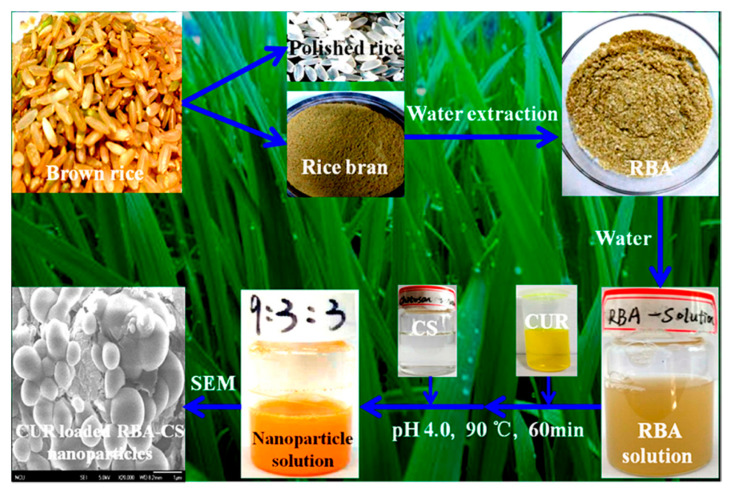
Schematic representation of the process used for the preparation of rice bran albumin and chitosan blend nanoparticles as carriers for curcumin. Reprinted with permission from ref. [135]. Copyright 2017 American Chemical Society.

Albumin present in rice bran was extracted and made into nanoparticles after blending these with chitosan, and were used as a carrier for curcumin. Ricebran albumin (RBA) was dispersed in water and later combined with chitosan dissolved in acetic acid. Curcumin dispersed in ethanol was added into the rice bran solution and the blend was later combined with the chitosan solution at pH 4, and constant stirring for 3 h at room temperature took place. The solution was later heated at 90 °C for 60 min to form the nanoparticles [135]. A schematic of the process used for the preparation of the composite nanoparticles is shown in Figure 21. The RBA-chitosan nanoparticles had an average diameter of 284 nm and a PDI of 0.23 which increased to 778 nm and 0.483–0.719 after the encapsulation of curcumin. An encapsulation efficiency of 93.6% was possible, and curcumin exhibited an initial burst and later a sustained release. The cytotoxicity of curcumin was enhanced when incorporated into the nanoparticles due to the higher stability and solubility of curcumin, which allows for higher levels of encapsulation [135]. In another study, highly water-stable proteins with a molecular weight of 31 kDa were extracted from Radix glycyrrhizae (licorice) and self-assembled (pH induced) nanoparticles with an average diameter of 206 nm. These nanoparticles were used to encapsulate aconitine, a herbal drug, and injected intraperitoneally into mice. Encapsulation with the rice proteins avoided the toxicity of aconitine due to detoxification, and hence these nanoparticles were suggested to be suitable for delivering toxic phytochemicals and herbal compounds [136].

Legumin, a protein found in cereals, was isolated from pea seed flour with a 95% purity. This protein was made into nanoparticles using co-acervation, and the co-acervates formed were hardened by crosslinking with glutaraldehyde. A yield of 27% nanoparticles with an average diameter of 243 nm, a PDI of 0.18, and a zeta potential of −18.7 were obtained. Nanoparticles were injected through intradermal immunization and the humoral and cell-mediated immune responses were recorded. The nanoparticles showed a cytostatic phenomenon and increased chromatin condensation [137].

Tea plants and, hence, the residues remaining after the preparation of tea, contain about 21–28% proteins, and 90% of these proteins are water insoluble. Ren et al. attempted to develop nanoparticles from tea water-insoluble proteins for their potential use as pickering emulsion stabilizers. The nanoparticles obtained had a diameter of 300 nm, a zeta potential of −30 mV, and ionic strength between 0–400 mM. Adding 2% nanoparticles to pickering emulsions led to substantial creaming stability due to their decreased droplet size, and avoided droplet flocculation or coalescence, suggesting these nanoparticles’ suitability for food applications [138].

Nanoparticles from uncommon plant proteins may have unique properties and distinct applications. However, the extraction of these proteins and the preparation of nanoparticles from these proteins, particularly in large quantities, are challenges. Unless specific needs arise, nanoparticles from the uncommon proteins will probably be only of academic interest.

## 6. Conclusions

Nanoparticles made from plant proteins have proven to be effective for the encapsulation and delivery of drugs and other biomolecules, bioimaging, increasing stability and the shelf-life of food, and for removing toxic components in water. Zein and gliadin are the predominant plant proteins used for developing nanoparticles, mainly because of their easy solubility in aqueous conditions. However, aqueous soluble components have been extracted from soy proteins and other legumes and made into nanoparticles. Anti-solvation is the most common approach for preparing the nanoparticles, but microfluidization, co-precipitation, and other techniques have also been used. Although it is difficult to accurately control the dimensions, the PDI of most of the nanoparticles are within the acceptable range. Studies that aimed to encapsulate and deliver biomolecules with the plant protein nanoparticles have shown that the bioavailability and stability to thermal treatment and gastro-intestinal conditions can be substantially improved. Protein nanoparticles have been internalized by the cytoplasm and show limited or no toxicity when injected in vivo. Similarly, adding protein nanoparticles into food emulsions has led to improvements in stability, rheological properties, and shelf-life. Despite these profound benefits, the practical applications of nanoparticles has been limited. Efforts to reduce cost, to increase production on a larger scale, and methods to develop nanoparticles from non-alcohol soluble proteins are necessary to make nanoparticles useable in large scale applications. Most of the studies on their controlled release applications have been done using drugs and pharmaceuticals and natural compounds through in vitro studies. Considerable increases in in vivo studies should take place to ensure the safety and efficacy of the protein nanoparticles. Similarly, research on the possibilities of using nanoparticles as carriers for bioagents, such as viruses and bacteria, should also be investigated. Further, nanoparticles that can be prepared in clinical settings for tailored applications would be ideal. For food applications, nanoparticles should be able to combine with various other biopolymers and should not adversely affect the nutritional value, texture, or functionality of food products. Plant protein-based nanoparticles offer immense potential but equally difficult constraints against their preparation and use. The quest to find ideal nanoparticles will continue more aggressively in the near future, and plant proteins promise to satisfy the requirements for the production and applications of nanoparticles in medicine, food and other areas.

## Figures and Tables

**Table 1:** List of polysaccharides used to stabilize zein nanoparticles intended for treating chronic degenerative diseases (CDD). Reprinted with permission from ref. [22]. Copyright 2018 Elsevier.

Zein-Polysaccharide Nanoparticles	Bioactive Compounds	CDD *	Activity	References
Zein-pectin	Omega-3 polyunsaturated fatty	Heart disease	Antihypertensive	[23]
Zein-pectin	Curcumin	Different diseases	Antioxidant, anti-inflammatory, antimicrobial. Improved thermal and UV resistance, ionic strength	[24]
Zein-pectin	Resveratrol	Cancer	Antioxidant and anticancer	[25]
Zein-NaCas-pectin **	Curcumin	Different diseases	Antioxidant	[26]
Zein-NaCas-pectin	Eugenol	Possible application nCDD	Antioxidant, antimicrobial	[27]
Zein-NaCas-pectin and Zein-NaCas-carboxymethyl cellulose zein-NaCas-gum arabic	Curcumin	Different diseases	Antioxidant	[28]
Zein-pectin-sodium alginate	Curcumin	Different diseases	Antioxidant	[29]
Zein-carboxymethyl chitosan	Vitamin D3	Chronic diseases	Prevention	[30]
Zein-carboxymethyl chitosan	Indole-3-carbinol and 3,30-diindolylmethane	Cancer	Anticancer	[31]
Zein-chitosan	Vitamin E (α-tocopherol)	Different diseases	Antioxidant	[32]
Zein-chitosan	Epigallocatechin gallate	Possible application in CDD	Antioxidant	[33]
Zein-carboxymethyl chitosan	Polyphenols and β-carotene	Cancer and Heart disease	Antioxidant	[34]
Zein-quaternized chitosan	Curcumin	Possible application in CDD	Antioxidant	[35]
Zein-Maillard conjugates *** and Zein-NaCas-dextran	Resveratrol	Health	Nutraceutical	[36]
Zein-shellac	Curcumin	Possible application in CDD	Antioxidant, anti-inflammatory and anticancer	[37]
Zein–sodium carboxymethyl cellulose	Paclitaxel	Cancer	Anticancer	[38,39]
Zein-chondroitin sulphate	Sorafenib	Cancer	Anticancer	[40]

CDD *: Chronic Degenerative Disease; NaCas **: sodium caseinate; Maillard conjugates ***: reaction NaCas-Dextran.

**Table 2:** Approaches used to improve the stability of zein nanoparticles. Reprinted with permission from ref. [21]. Copyright 2018 Frontiers.

Applications of Nanoparticles/Payload Used	Strategies to Improve Stability	Stability Results	References
5-fluorouracil	Formulation stored at 4 °C	Coating increased EE, nanoparticlesaggregated at low concentration of emulsifiers.	[41]
Thymol	Coated with caseinate and chitosan	Coated particles were stable at pH 3 to 8.	[42]
Mint oil	Coated with gum arabic	Uncoated particles released oil faster	[20]
Thymol and carvacrol	Formulation stored at 4 °C	Nanoparticles precipitated after 2 months at 20 °C but not at 4 °C	[43]
Resveratrol	Coated with sodium caseinate	Coating improved stability for up to 28 days	[36]
Lutein	Coated with lecithin and Pluronic	Coating improved stability for up to 30 days	[44]
Hollow zein	Thermal treatment in thermostatic water bath	Treatment at 75 °C for 15 min resulted in lower diameter and PDI	[45]
Hollow zein	Coated with carrageenan	Coating maintained constant diameter for 30 days at pH between 5.25 and 6.75.	[46]
Resveratrol	Coated with pectin	Stability was dependent on pectin concentration	[47]
Resveratrol	Coated with chitosan	Coating improved gastrointestinal stability and mucoadhesive properties	[48]
Anarcardic acid	Dimetilglyoxim	381.6 nm particles were stable with negative ζ potential and antimicrobial activity, pH, salt and thermal stability, improved bioaccessibility and antioxidant activity.	[49]
Curcumin-Natamycin	70% alginate and 30% fish gelatinCarboxymethyl chitosan	Remained stable and were resistant to neutral pH (6.0–8.0), ion strength (0–100 mM) and long-term storage (30 days).	[50]
Epithelial cells—293	Chondroitin sulfate as a stabilizer.	-Stable nanoparticles between pH 3 and 8	[51]

**Table 3:** Properties of zein nanoparticles and their ability to sorb curcumin. Reprinted with permission from ref. [60]. Copyright 2020 Elsevier.

Sample	Particle Size, nm		Poly Dispersity Index (PDI)		Zeta Potential, mV		Encapsulation Efficiency, %		Loading Capacity, %
	before	after	before	after	before	after	before	after	
Zein-curcumin	183 ± 4	2031 ± 177	0.272 ± 0.003	0.369 ± 0.091	−17.7 ± 0.2	−2.2 ± 0.7	67 ± 1.3	4.6 ± 0.9	4.6 ± 0.94
	358 ± 11	876 ± 12	0.234 ± 0.002	0.331 ± 0.021	−25.6 ± 0.4	−19.1 ± 2.0	79.4 ± 2.1	4.9 ± 1.0	4.9 ± 1.0
	230 ± 24	209 ± 5	0.286 ± 0.009	0.213 ± 0.029	−35.3 ± 0.6	−39.3 ± 1.0	76.1 ± 0.9	4.8 ± 1.2	4.8 ± 1.2
	239 ± 1	226 ± 11	0.314 ± 0.004	0.196 ± 0.025	−41.7 ± 0.7	−41.5 ± 0.5	72.8 ± 1.5	4.7 ± 0.9	4.7 ± 0.9

**Table 4:** Properties of gliadin and gliadin-gelatin nanoparticles produced using the electrospray technique. Reprinted with permission from ref. [97]. Copyright 2012 American Chemical Society.

Nanoparticle	Average Size, nm	Zeta Potential, mV	Drug Loading, %
7% gliadin	218.66 ± 5.1	18.46 ± 8.3	72.02 ± 5.6
7% gliadin, 4% gelatin	398.56 ± 4.2	19.00 ± 3.6	64.23 ± 8.9
7% gliadin, 8% gelatin	450.10 ± 9.7	14.20 ± 3.7	52.77 ± 12.6

**Table 5:** Properties of soy protein nanoparticles before and after loading with curcumin. Reprinted with permission from ref. [100]. Copyright 2012 American Chemical Society.

Curcumin/Protein Ratio	Encapsulation Efficiency, %	Loading Efficiency, %	Particle Size, nm	Zeta Potential, mV
Pure proteins	-	-	201.5 ± 9.2	−36.8 ± 1.0
1% curcumin	97.2 ± 2.0	1.1 ± 0.1	220.1 ± 17.8	−36.0 ± 2.1
2% curcumin	81.2 ± 1.2	1.7 ± 0.1	252.6 ± 13.4	−35.2 ± 0.8
3% curcumin	52.8 ± 3.0	2.7 ± 0.2	286.7 ± 10.1	−34.5 ± 1.4

**Table 6:** Properties of soy protein nanoparticles (prepared using 1 or 8% solution) before and after treating with glutaminase. Reprinted with permission from ref. [107]. Copyright 2018 Elsevier.

Parameter	Curcumin	Untreated Soy Protein		Enzyme Treated Soy Protein	
		1%	8%	1%	8%
Encapsulation Efficiency, %	-	96.7 ± 0.27	97.6 ± 0.21	97.3 ± 0.30	98.4 ± 0.07
Loading amount, µg/mg	-	36.2 ± 0.93	25.7 ± 0.56	101 ± 2.41	34.9 ± 0.06
Diameter, nm	No Yes	86.4 ± 1.05 105 ± 0.76	402 ± 0.15 547 ± 0.38	113 ± 1.27 142 ± 1.05	523 ± 0.78 692 ± 2.10
Zeta potential, mV	No Yes	−31.1 ± 0.85 −38.4 ± 0.27	−32.3 ± 1.27 −37.7 ± 1.81	−8.5 ± 0.82 −30.9 ± 1.61	−11.1 ± 0.16 −30.6 ± 1.13
Hydrophobicity	No Yes	3472 ± 6.51 2779 ± 0.99	3101 ± 1.56 2926 ± 2.55	3827 ± 4.17 2926 ± 2.55	4176 ± 2.47 2534 ± 3.61

**Table 7:** Influence of various parameters on the size and zeta potential of soy protein nanoparticles. Reprinted with permission from ref. [111]. Copyright 2012 Elsevier.

CaCl_2_ (mM)	pH	Diameter, nm	PDI	Zeta Potential, mV	pH	Size, nm		Zeta Potential, mV	
						2.5 mM	5 mM	2.5 mM	5 mM
2.5	7	60 ± 3	0.28 ± 0.00	−11.7 ± 0.3	2	33.9 ± 2.4	82.8 ± 1.3	16.9 ± 0.9	17.3 ± 2.3
-	8	28 ± 1	0.27 ± 0.00	−14.3 ± 0.7	3	45.9 ± 5.6	99.4 ± 1.4	18.7 ± 0.6	19.3 ± 1.5
5	8	101 ± 2	0.29 ± 0.00	−8.8 ± 0.6	7	100.8 ± 14.7	146.0 ± 13.3	−10.3 ± 0.4	−9.5 ± 2.1
-	9	71 ± 3	0.23 ± 0.03	−11.0 ± 0.3	8	32.7 ± 7.2	119.0 ± 35.1	−15.8 ± 1.1	−10.8 ± 2.3
10	9	179 ± 3	0.27 ± 0.00	−7.5 ± 0.6	9	29 ± 10.2	76.0 ± 0.5	−19.1 ± 0.2	−11.4 ± 1.7

**Table 8:** Properties of peanut protein nanoparticles with variations in pH and the amount of Ca2+ ions. Reprinted with permission from ref. [130]. Copyright 2020 Elsevier.

Parameter	pH	CaCl2 Concentration						
		0 mM	1 mM	2.5 mM	3.5 mM	5 mM	7.5 mM	10 mM
Z-ave (nm)	7	134.1 ± 0.5 ^b^	125.5 ± 0.9 ^a^	173.1 ± 1.8 ^c^	429.2 ± 17.8 ^d^	5470.7 ± 1032.1 ^e,^^f^	4474.0 ± 400.0 ^e^	5870.7 ± 355.6 ^f^
	8	142.4 ± 0.4 ^b^	138.3 ± 1.2 ^a^	157.9 ± 1.9 ^c^	191.2 ± 4.2 ^d^	455.5 ± 8.6 ^e^	6414.0 ± 809.8 ^f^	5873.3 ± 771.5 ^f^
	9	143.0 ± 0.3 ^a^	146.2 ± 1.5 ^b^	176.6 ± 2.5 ^c^	202.3 ± 6.6 ^d^	194.8 ± 1.5 ^d^	247.2 ± 1.3 ^e^	799.2 ± 36.4 ^f^
Turbidity	7	0.432 ± 0.015 ^a^	0.494 ± 0.009 ^b^	1.215 ± 0.021 ^c^	3.072 ± 0.045 ^d^	3.298 ± 0.035 ^e^	3.307 ± 0.043 ^e^	3.303 ± 0.055 ^e^
	8	0.416 ± 0.021 ^a^	0.478 ± 0.014 ^b^	0.802 ± 0.017 ^c^	1.268 ± 0.023 ^d^	2.790 ± 0.037 ^e^	3.232 ± 0.030 ^f^	3.260 ± 0.042 ^f^
	9	0.408 ± 0.012 ^a^	0.428 ± 0.011 ^a^	0.615 ± 0.010 ^b^	0.921 ± 0.010 ^d^	0.892 ± 0.015 ^c^	1.861 ± 0.022 ^e^	3.065 ± 0.037 ^f^
PDI	7	0.245 ± 0.004 ^a^	0.265 ± 0.002 ^b^	0.257 ± 0.004 ^b^	0.573 ± 0.094 ^c^	0.421 ± 0.183 ^c^	0.422 ± 0.066 ^c^	0.458 ± 0.070 ^c^
	8	0.259 ± 0.011 ^a^	0.250 ± 0.010 ^a^	0.363 ± 0.015 ^b^	0.372 ± 0.037 ^b^	0.410 ± 0.092 ^b^	0.633 ± 0.075 ^c^	0.597 ± 0.036 ^c^
	9	0.245 ± 0.008 ^a^	0.260 ± 0.005 ^a^	0.309 ± 0.034 ^b^	0.474 ± 0.058 ^c^	0.423 ± 0.007 ^c^	0.442 ± 0.008 ^c^	0.997 ± 0.006 ^d^
Zeta-potential (mV)	7	−21.90 ± 2.15 ^f^	−16.70 ± 1.78 ^e^	−13.90 ± 0.75 ^d^	−11.40 ± 0.80 ^c^	−8.39 ± 0.41 ^b^	−4.98 ± 0.54 ^a^	−4.64 ± 0.45 ^a^
	8	−26.27 ± 1.40 ^e^	−23.90 ± 1.15 ^d^	−21.10 ± 1.97 ^d^	−16.67 ± 1.36 ^c^	−14.53 ± 1.36 ^c^	−10.54 ± 0.55 ^b^	−8.52 ± 0.83 ^a^
	9	−29.17 ± 2.20 ^e^	−25.77 ± 1.31 ^e^	−19.63 ± 1.68 ^d^	−15.87 ± 1.00 ^c^	−13.17 ± 1.31 ^c^	−10.69 ± 0.97 ^b^	−7.72 ± 0.85 ^a^

Data points with same superscript alphabets are not statistically significant.

**Table 9:** Conditions used and properties of nanoparticles (NPs) obtained from walnut protein isolates (WPI) through the electrospraying approach. Reprinted with permission from ref. [131]. Copyright 2021 Springer Nature.

Sample	Unloaded NPs	Curcumin Loaded NPs
Concentration, % *w*/*w*	3.5	3.5
Curcumin:WPI ratio, % *w*/*w*	-	0.111111111
Apparent viscosity mPaS	12.25 ± 3.35	6.35 ± 0.32
Electrical conductivity (μS/cm)	177.12 ± 7.14	133.5 ± 6.13
Zeta potential (mV)	−14.6 ± 2.01	−17.7 ± 0.94
Voltage (kV)	20	20
Tip to collector distance (cm)	17.5	17.5
Flow rate (mL/h)	0.09	0.09
Nozzle diameter	18	18

## Data Availability

All the data is available within the manuscript.
